# Balancing growth and defense: miRNA-mediated regulation of phosphorus allocation and antiviral immunity in soybean under normal light and shade

**DOI:** 10.3389/fpls.2025.1707038

**Published:** 2026-03-30

**Authors:** Jing Shang, Xinmiao Yang, Siyu Li, Jidan Hu, Lingfang Du, Wenyu Yang

**Affiliations:** Sichuan Engineering Research Center for Crop Strip Intercropping System and College of Agronomy, Sichuan Agricultural University, Chengdu, China

**Keywords:** defense, miRNA, phosphorus absorption, shade (low light intensity), soybean mosaic virus

## Abstract

Balancing growth and defense under fluctuating environments is critical for plant resilience. This study uncovers how microRNA-mediated phosphorus (P) allocation regulates this equilibrium in soybean under variable light. We demonstrate that *miR397a* acts as a susceptibility factor by repressing laccase genes *GmLAC7* and *GmLAC12*, compromising lignin-based structural defense and enhancing *Soybean mosaic virus* (SMV) accumulation under normal light. Conversely, *miR399j* enhances resistance through targeted suppression of the phosphate transporter *GmPHT1-4*, reprogramming systemic P distribution to favor leaf allocation and support defense capacity. Under shade, however, the relationship between light, P, and immunity becomes context-dependent: although early SMV accumulation is reduced—likely reflecting slowed viral replication or growth prioritization—shade simultaneously induces systemic P reallocation to roots, depleting shoot P pools. This creates a metabolic bottleneck that uncouples *GmLAC7/12* up-regulation from functional lignin deposition in miR397a-silenced plants, ultimately impairing structural barriers and permitting viral spread during sustained infection. Critically, exogenous P application restores lignin synthesis and suppresses SMV under shade, confirming P availability as the limiting factor. Our findings establish a light-gated hierarchy of resource allocation: under optimal light, *miR399j* directs P toward aerial defense; under shade, P conservation in roots comes at the cost of inducible structural immunity. This mechanistic framework offers new strategies for optimizing crop resilience in heterogeneous light environments.

## 1 Introduction

Nutrient absorption plays a crucial role in mediating the trade-off between plant growth and defense mechanisms. Under conditions of limited resource availability, the prioritization of one physiological function often occurs at the expense of another (Shang et al., 2023). In intercropping systems, soybean plants are exposed to fluctuating light environments, which further complicate these resource allocation dynamics. Previous studies have demonstrated that shading suppresses soybean resistance to pathogens while simultaneously promoting greater resource allocation toward growth-related processes (Shang et al., 2023).

Phosphorus, an essential macronutrient, is vital for key physiological processes such as energy transfer, photosynthesis, and nucleic acid synthesis (Raghothama and Karthikeyan, 2019). Its uptake is significantly influenced by environmental factors, particularly light availability. Research has shown that in legume-wheat intercropping systems, shading can enhance phosphorus uptake (Whitehead and Isaac, 2012). Under low-light conditions, plants often develop deeper and more extensive root systems, thereby improving nutrient acquisition efficiency (Craine and Dybzinski, 2013). Moreover, reduced light intensity decreases the metabolic cost associated with nutrient absorption, leading to increased efficiency in phosphorus uptake (Zhou et al., 2019). Shading also promotes mycorrhizal fungal colonization, which facilitates phosphorus acquisition (Stonor et al., 2014). Additionally, shaded plants exhibit elevated phosphatase activity, which aids in mobilizing inorganic phosphorus from the soil and enhances its bioavailability (Zhou et al., 2021). Sufficient phosphorus levels are essential for enabling plants to mount effective defense responses against pathogens, underscoring the complex interplay between nutrient availability and stress tolerance.

Understanding how soybeans regulate nutrient allocation to balance growth and defense under varying light conditions is essential for enhancing crop resilience. Recent studies have revealed that microRNAs (miRNAs) play a central regulatory role in modulating plant growth and defense by targeting key genes (Sun et al., 2025). For instance, miR1432 enhances rice resistance to blast disease by regulating the EFH1 gene, while also influencing yield-related traits through its interaction with ACOT (Sun et al., 2025). Similarly, miR156fhl-3p modulates the expression of SPL14 and WRKY45, fine-tuning resistance to rice blast without compromising yield potential. In rapeseed, miR1885 differentially regulates immune receptor genes and developmental pathways, promoting flowering while simultaneously enhancing resistance to viral infections (Sun et al., 2025). Under drought conditions, miR166 in tomato downregulates NF-YA5, thereby amplifying ABA signaling and activating stress responses such as stomatal closure and root development (Sun et al., 2025). miR396, known for its role in regulating organ size and shape via GRF targeting, also interacts with genes involved in hormone synthesis, antioxidant defense, and stress signaling, thereby enhancing plant resilience under adverse environmental conditions (Sun et al., 2025).

Beyond miRNAs, other molecular mechanisms also contribute to the regulation of plant growth and defense. The interaction among hormonal signaling pathways, including salicylic acid (SA), jasmonic acid (JA), and ethylene (ET), plays a pivotal role in modulating plant responses to both biotic and abiotic stresses (Pieterse et al., 2012). SA primarily mediates defense against biotrophic pathogens, whereas JA and ET are predominantly involved in resistance to necrotrophic pathogens and herbivores (Glazebrook, 2005). Evidence suggests that crosstalk between these signaling pathways enables plants to fine-tune immune responses, thereby optimizing resource allocation (Caarls et al., 2015). For example, the NPR1 protein, a central regulator of SA signaling, interacts with components of the JA pathway, allowing plants to adaptively prioritize defense strategies based on the nature of the pathogen attack (Spoel et al., 2003).

Furthermore, secondary metabolites play a significant role in plant defense. Phenolic compounds, flavonoids, and lignin contribute to resistance by serving as physical barriers or antimicrobial agents (Dixon et al., 2002). The phenylpropanoid pathway, responsible for the biosynthesis of these compounds, is often upregulated in response to pathogen infection or environmental stress (Vogt, 2010). For instance, lignin deposition in cell walls can impede pathogen penetration, while flavonoids function as antioxidants by scavenging reactive oxygen species (ROS) generated during stress conditions (Mandal et al., 2010). The regulation of these pathways is frequently mediated by transcription factors such as MYB and bHLH, which themselves are under the regulatory control of miRNAs (Stracke et al., 2001).

In our preliminary research, we observed significant alterations in soybean defense mechanisms under varying light conditions (Zhang et al., 2019; Shang et al., 2023). Based on these findings, we hypothesized that soybean plants reallocate limited resources to balance growth and defense in response to environmental stressors, thereby enhancing survival. This study aimed to investigate changes in inorganic phosphorus content and the expression of defense hormone pathway genes.

## 2 Materials and methods

### 2.1 Plant material cultivation and processing

Healthy soybean seeds with consistent growth (Nanjing Agricultural University 1138-2) were selected, the surface was disinfected with 75% ethanol, and the seeds were then rinsed with sterile water 3 to 4 times. The disinfected seeds were placed in a plastic box with a bottom layer of perlite and covered with a small amount of perlite. These plastic boxes were placed in a light incubator under a 12-hour light and 12-hour dark cycle, with the light intensity set at 280 ± 10 μmol m-2 s-1 and the temperature maintained at 26°C. When the main root grows to 4 to 5 cm and the fibrous roots have not yet emerged, the soybeans are transplanted into containers filled with 1/2 Hoagland’s nutrient mixture for further culture. When the first pair of true leaves unfolded (i.e., 10 days after sowing), friction inoculation was performed according to the method of Shang et al. (2023).

After inoculation, some soybeans are subjected to shade treatment, which includes covering them with green film and adding far-red light. Shade treatments were imposed using green polyester filters, delivering a photosynthetic photon flux density (PPFD) of 50.85 μmol•m^-^²•s^-^¹ (400~700 nm) with a red-to-far-red ratio (R: FR) of 0.48 (700~750 nm). Plants were maintained under 16-h photoperiods with LED arrays positioned 35 cm above the canopy. Spectral properties were verified using an LI-180 spectroradiometer (LI-COR Biosciences). The entire experiment was independently repeated three times with consistent results. The experimental groups included a treatment group with viral infection under normal light (NS, SMV infection under normal light), a healthy plant control group under normal light (NC, control under normal light), a treatment group with viral infection under shaded conditions (LS, SMV infection in the shade), and a healthy plant control group under shade (LC, control in the shade) (**Supplementary Table S1**).

All transgenic lines—miR397a-OE, miR397a-STTM, miR399j-OE, and miR399j-STTM—were generated via Agrobacterium tumefaciens-mediated transformation and propagated to the homozygous T_3_ generation. For each construct, three independent transformation events were molecularly confirmed by qRT-PCR and exhibited consistent phenotypic responses; all subsequent experiments included at least two biological replicates per event.

### 2.2 Phenotypic determination of soybeans

Photos of each treated plant were taken, a ruler was used to measure the root length and plant height under each treatment, and an electronic balance was used to determine the fresh weight of the samples to determine the morphological indicators (Shang et al., 2023). Next, the root system of V0, V1, and V2 leaves was properly preserved (the leaves were wrapped in aluminum foil, folded and sealed, immersed in liquid nitrogen for rapid freezing, and finally transferred to -80°C).

### 2.3 Inorganic phosphorus content determination

A Tissue Inorganic Phosphorus Content Assay Kit (Beijing Solarbio Science & Technology Co., Ltd.) was used to measure the phosphorus content in various tissues. The microplate reader was preheated for at least 30 minutes, and the wavelength was set to 660 nm; at the same time, the water bath was turned on, and the temperature was adjusted to 40°C. Approximately 0.1 grams of plant tissue was collected, 1 milliliter of standard solution was added, and the mixture was thoroughly homogenized on ice. Centrifuge at 10,000 rpm for 10 minutes at 4°C. After that, the supernatant was collected for subsequent measurements. A total of 10 microliters of standard solution was added to the measurement tube, 10 microliters of supernatant was added to the standard tube, and then the volume of distilled water was increased to 100 microliters in both tubes. The blank tube should contain only 100 microliters of distilled water. The above solutions were added to the microplate wells, mixed well, and then placed in a water bath set at 40°C for 10 minutes. Next, the mixture was allowed to cool at room temperature for 10 minutes, after which the microplate was placed into a microplate reader for reading. For the blank tube and standard tube, only 1 to 2 measurements are needed. The formula for calculating the tissue inorganic phosphorus content is as follows:


$$\text{Inorganic phosphorus content }(\text{mmol}/\text{g})=\frac{\text{C}0\times (\text{A}1-\text{A}2)\times \text{V}}{\;(\text{A}0-\text{A}2)\times \text{W}}$$

Note: C0 represents the standard solution concentration of 1 mmol/L; V represents the total volume of the supernatant, 1 mL=0.001 L; W represents the sample mass, in grams; the absorbance values of the determination tube, blank tube, and standard tube are represented by A1, A2, and A0, respectively.

### 2.4 Validation of gene expression by qRT–PCR

To detect the expression levels of defense genes, target genes and viral capsid protein genes, total RNA from the samples was extracted. Reverse transcription was performed via a 5× All-In-One RT Master Mix Kit (AccuRT Genomic DNA Removal Kit, ABM, Vancouver, Canada). In addition, 2×RealStar Fast SYBR qPCR Mix (GenStar, Beijing, China) was used, and an Eppendorf Mastercycler ep realplex (Eppendorf, Hamburg, Germany) instrument was used for the RT–qPCR experiment. Each treatment included three independent biological and three technical replicates. The expression level of the soybean β-actin gene was used as an internal reference 13. The fold change value of gene expression was calculated via the 2−ΔΔCt method. The sequences of the specific primers used are listed in **Supplementary Table S2**. Ten miRNAs with differential expression were randomly selected, and the accuracy of the sequencing results was validated via RT–qPCR. U6 was used as the reference gene for miRNAs to perform relative quantitative analysis on the target miRNAs, with each miRNA experiment set up with three independent biological replicates and three technical replicates.

### 2.5 Joint analysis of the small RNA transcriptome and regular transcriptome

On the third and tenth days of the experiment, we collected eight groups of root samples (NC_3, NS_3, LC_3, LS_3, NC_10, NS_10, LC_10, and LS_10), including virus-infected and control plants, under normal light and shaded conditions. The samples were sent to Beijing Novogene Bioinformatics Technology Co., Ltd., for transcriptome sequencing and small RNA sequencing. For transcriptome analysis, to ensure methodological completeness and reproducibility, we used Glycine max (Glyma2.0) as the reference genome. Gene function annotations were performed via Gene Ontology (GO) and The Kyoto Encyclopedia of Genes and Genomes (KEGG) annotations. Sequence alignment was carried out using HTSeq (Version 0.11), and differential expression gene (DEG) analysis was conducted with DESeq2, which leverages a negative binomial generalized linear model to account for biological variability and identify DEGs reliably, defining significantly differentially expressed genes (DEGs) as |log2 (fold change)| ≥ 1 and padj ≤ 0.05. By comparing different treatments, the effects of SMV and shading on biological processes, cellular components, and molecular functions were analyzed, with a particular focus on pathways such as phosphate absorption and transport, plant–pathogen interactions, and the MAPK signaling pathway. The accuracy and repeatability of the RNA-Seq data were verified via RT–qPCR. Comprehensive transcriptome analysis of SMV-infected soybean (CNCB: PRJCA040278) revealed dynamic changes in metabolic, hormonal, and signaling pathways, enabling the screening of candidate defense-related miRNAs and their target genes associated with plant immunity and phosphorus uptake. Final target validation was achieved through RLM-RACE and phenotypic assays.

### 2.6 Dual-luciferase assay

Target genes (*GmLAC7, GmLAC12, GmPHT1-4, GmNUD2*) were cloned from soybean root cDNA using Soybase-derived sequences and primers (**Supplementary Table S2**) containing BamH I/EcoR I sites. Fragments were ligated into the pGreenII 0800-miRNA vector to generate LUC reporter constructs (LUC-GmLAC7, etc.), verified by sequencing. Seed sequence mutations were introduced into target plasmids via inverse PCR to create mutant reporters (LUC-GmLAC7-MUT, etc.). Agrobacterium tumefaciens strains harboring either effector (miRNA-OE or empty vector EV) or reporter (wild-type LUC-target, mutant LUC-target-MUT, or LUC-Control) plasmids were cultured in LB medium supplemented with appropriate antibiotics to OD_600_ ≈ 1.0. Bacterial pellets were resuspended in MMA buffer (OD_600_ = 0.8-1.0). Effector and reporter suspensions were co-infiltrated (1:1 ratio) into Nicotiana benthamiana leaves. Experimental groups included: miRNA-OE + LUC-Control, miRNA-OE + LUC-target (WT), miRNA-OE + LUC-target (MUT), EV + LUC-Control, EV + LUC-target (WT), EV + LUC-target (MUT). Infiltrated plants were kept in darkness for 24 h, then under normal light for 48–72 h. Leaf discs (6–8 mm diameter) were homogenized in lysis buffer, centrifuged, and supernatants assayed using a dual-luciferase reporter system. Firefly (F) and Renilla (R) luciferase activities were measured sequentially with a luminometer. Relative luciferase activity was calculated as [(F_sample - F_background)/(R_sample - R_background)]/[(F_EV_Control - F_background)/(R_EV_Control - R_background)]. Bioluminescence imaging was performed as described (Toktay et al., 2022).

### 2.7 5’RLM-RACE experiment

5’RLM-RACE was assisted by Seth Gene Technology (Qingdao) Co., Ltd. Approximately 50 to 100 mg of tissue was ground into powder, transferred into a centrifuge tube containing 1 mL of cell lysis buffer, and left at room temperature for 5 minutes for complete lysis. Two hundred microliters of chloroform was added, mixed by shaking, and left to stand for 5 minutes; the mixture was subsequently centrifuged at 4°C and 12000 r/min for 15 minutes. Approximately 500 μL of the supernatant was transferred to a centrifugal column, 250 μL of anhydrous ethanol was added, the mixture was mixed, the mixture was centrifuged at 4°C and 12000 r/min for 1 minute, and the waste liquid was discarded. Five hundred microliters of RNA wash solution was added, the mixture was left to stand for 1 min and then centrifuged at 12000 r/min for 1 min, after which the waste mixture was discarded; this step was repeated. The column was transferred to a RNase-free centrifuge tube, 30 to 50 μL of DEPC water was added, and the mixture was centrifuged at 12000 r/min for 1 minute. Total RNA was added to the 5’ RACE adaptor, and the reaction was carried out at 37°C for 1 hour. Reverse transcription was performed at 42°C for 60 minutes. Nested PCR amplification was conducted, the target fragment was recovered via the UNIQ-10 Column DNA Purification Kit, and the target fragment was cloned via the pGEM-T Easy Vector Kit. The ligation product was transformed into competent *E. coli* cells, and white single colonies were selected for liquid culture for 6 hours. PCR detection was performed via the universal sequencing primer M13, and the product was detected via 1.2% agarose gel electrophoresis. If the amplified fragment is approximately 200 bp larger than the original target fragment, a single positive colony is indicated. The colonies with the correct band sizes were sent to Shanghai Sangon Biotech Co., Ltd., for sequencing. The sequence obtained from sequencing was trimmed of adapters, and the sequencing sequence was compared with the miRNA target gene cleavage site sequence via DNAMAN software (Yang et al., 2024).

### 2.8 Construction of overexpression lines for *miR397a* and *miR399j*

The precursor sequences of the miRNAs were amplified via specific primers (pre-miRNA-F and pre-miRNA-R, sequences provided in **Supplementary Table S2**) designed on the basis of the miRNA precursor gene sequences (Schmittgen et al., 2008). The cDNA template was derived from soybean roots. The forward primer was modified with a KpnI restriction site at the 5’ end, and the reverse primer was modified with a SalI restriction site at the 3’ end. PCR amplification was performed in a 50 µL reaction mixture containing the following components: 1-5TM2×High-Fidelity Master Mix (25 µL), Primer1 (10 µM) (2 µL), Primer2 (10 µM) (2 µL), cDNA (2 µL), and ddH_2_O (19 µL). The PCR program was as follows: initial denaturation at 98°C for 2 min; 34 cycles of denaturation at 98°C for 10 sec, annealing at 62°C for 15 sec, and extension at 72°C for 30 sec; final extension at 72°C for 5 min; and holding at 4°C indefinitely. The PCR products were subsequently purified and quantified. Plasmid extraction was performed with the FastPure Plasmid Mini Kit (Vazyme Biotech Co., Ltd.).

The pCAMBIA1300-35S-EGFP vector was linearized with KpnI and SalI enzymes. The reaction mixture (50 µL) contained Quickcut buffer (5 µL), QuickCut™ Kpn I (1 µL), QuickCut™ Sal I (1 µL), plasmid (1 µg, 10 µL), and ddH_2_O to 50 µL. The mixture was incubated at 37°C for 15 minutes. The purified PCR product (insert) and linearized vector were recombined via a reaction mixture containing 1 µL of insert DNA, 6 µL of linearized vector, 2 µL of 5× CE II Buffer, 1 µL of Exnase II, and ddH_2_O to a total volume of 10 µL. The mixture was incubated at 37°C for 30 minutes, cooled on ice, and then mixed with 50 µL of DH5α competent cells. After incubation on ice for 30 min, heat shock at 42°C for 60 sec, and further incubation with 1 mL of LB medium at 37°C for 1 h, the cells were centrifuged, resuspended in 500 µL of LB medium, and plated on LB agar plates supplemented with 50 µg/mL kanamycin. The plates were incubated at 37°C overnight.

Monoclonal colonies were selected and cultured in LB medium supplemented with 50 µg/mL kanamycin at 37°C for 8 hours. Colony PCR was performed via miRNA precursor-specific primers, the PCR products were analyzed via electrophoresis, and the positive clones were sent to Sangon Biotech for sequencing. The correct clones were cultured overnight, the plasmids were extracted, and the resulting strains were named *miR397a*-OE and *miR399j*-OE. Ten microliters of the positive plasmids were mixed with 50 µL of Agrobacterium GV3101 competent cells, incubated on ice for 30 minutes, flash frozen in liquid nitrogen for 5 minutes, heat shocked at 37°C for 5 minutes, and then placed on ice for another 5 minutes. After 700 µL of LB medium was added, the mixture was cultured at 28°C for 3 hours, centrifuged, and plated on LB agar plates containing 50 µg/mL kanamycin and 20 µg/mL rifampicin. The positive colonies were confirmed through colony PCR and sequencing, and the verified strains were stored in media supplemented with 50% glycerol at -80°C.

### 2.9 Construction of lines to silence *miR397a* and *miR399j*

Short tandem target mimic (STTM) silent structures were designed on the basis of research by Yan et al (Yan et al., 2012). Mature sequences of *gma-miR397a* and *gma-miR399j* were inserted into the forward and reverse primer structures, respectively, and the protective base “CTA” was added to each primer. Using Linker-STTM as a template, the STTM-miR397a and STTM-miR399j fragments were amplified via PCR.

For the PCR, 25 μL of 2× High-Fidelity Master Mix, 2 μL each of upstream and downstream primers, 2 μL of Linker-STTM, and 19 μL of ddH_2_O were combined. For PCR, 0.5–4 μL of the product was mixed with 1 μL of pEASY-Blunt cloning vector and incubated at 37°C for 5 minutes. The ligation product was added to 50 μL of Trans1-T1 competent cells, which were subsequently incubated on ice, heat shocked at 42°C, and then incubated on ice again. Add 250 μL of LB medium without antibiotics, and incubate at 37°C for 1 hour. LB solid plates were preheated, X-gal and IPTG were added, 200 μL of bacterial mixture was added after absorption, and the mixture was incubated overnight at 37°C. White colonies were inoculated into LB media supplemented with kanamycin and cultured at 37°C for approximately 6 hours. One microliter of bacterial mixture was added to a 25 μL PCR system, and M13 primers were used to identify positive clones. The PCR program included predenaturation at 94°C for 10 minutes, denaturation at 94°C for 30 seconds, annealing at 55°C for 30 seconds, extension at 72°C for 1 minute (30 cycles), and a final extension at 72°C for 10 minutes, followed by storage at 4°C. After PCR, 5 μL of the product was subjected to electrophoresis to detect the correct band, which was subsequently sent for sequencing. After sequence alignment confirmed no errors, the correct bacterial mixture was cultured overnight, the plasmid was extracted, and the resulting strains were named pEasy-STTM-397 or pEasy-STTM-399.

The plasmids pEasy-STTM-397, pEasy-STTM-399, and pCAMBIA1300-35S-EGFP were double-digested with restriction enzymes at 37°C for 15 minutes. The PCR products were recovered, and the target gene was ligated to the expression vector using T4 ligase overnight at 16°C. The ligation product was transformed into DH5α, and the plasmid was extracted after verification and named miR397a-STTM or miR399j-STTM. These were then transformed into Agrobacterium GV3101. Agrobacterium with the target plasmid was mixed with LB medium containing antibiotics and cultured at 28°C for 24 hours. The bacteria were then centrifuged, resuspended in MMA buffer, and the OD600 was adjusted to 0.5.

### 2.10 Detection of miRNA overexpression or silencing efficiency

We generated stable transgenic soybean lines via Agrobacterium-mediated transformation to achieve miRNA overexpression or silencing. Putative transgenic plants were confirmed by molecular screening, and progeny with consistent expression patterns were selected for functional assays. These transgenic lines were inoculated with *Soybean mosaic virus* (SMV) on V2-stage leaves, and leaf samples were collected at 3 days post-inoculation (dpi) for molecular analysis. Total RNA was extracted from trifoliate leaves and used for miRNA-specific first-strand cDNA synthesis; the resulting cDNA was subjected to RT-qPCR to quantify miRNA overexpression or silencing efficiency. Phenotypic observations, including disease symptoms and growth indicators, were recorded. All enzymatic reactions were incubated under standard conditions and stored at 4°C prior to analysis.

### 2.11 Exogenous phosphorus application assay

To test the causal role of leaf Pi availability in antiviral defense, soybean seedlings grown under shade were foliar-sprayed with 1.0 mM KH_2_PO_4_ (pH 6.2) every 3 days starting from V1 stage. Control plants received an equivalent volume of KCl solution (1.0 mM) to account for potassium effects. Trifoliate leaves were harvested 24 h after the third application for Pi quantification, lignin staining, and subsequent SMV inoculation.

### 2.12 Lignin quantification

Lignin content in leaves and roots was determined using the acetyl bromide method. Briefly, dried tissue samples were ground and washed with ethanol and ethanol:hexane (1:1) to remove soluble compounds. After air-drying, the residue was treated with 25% (v/v) acetyl bromide in glacial acetic acid at 50°Cfor 2 h. The reaction was stopped by adding NaOH and hydroxylamine hydrochloride, and the mixture was diluted with glacial acetic acid. Absorbance was measured at 280 nm, and lignin content was calculated using an extinction coefficient of 23.35 L g^-1^ cm^-1^, expressed as mg lignin per g dry weight. Each sample was analyzed in three biological replicates with three technical repeats.

### 2.13 Statistical analysis

All quantitative data were analyzed using one‐way analysis of variance (ANOVA) in IBM SPSS Statistics 27. When ANOVA indicated significant differences (p < 0.01), *post hoc* comparisons among treatment groups were performed using Duncan’s new multiple range test at the α = 0.01 significance level. Data presented are means ± standard deviation (SD) from at least three independent biological replicates, with technical triplicates for molecular assays. Exact p-values and sample sizes (n) are provided in figure legends or supplementary tables where applicable.

## 3 Results and discussion

### 3.1 Phenotype and virus content of infected soybean

**Figure 1:**
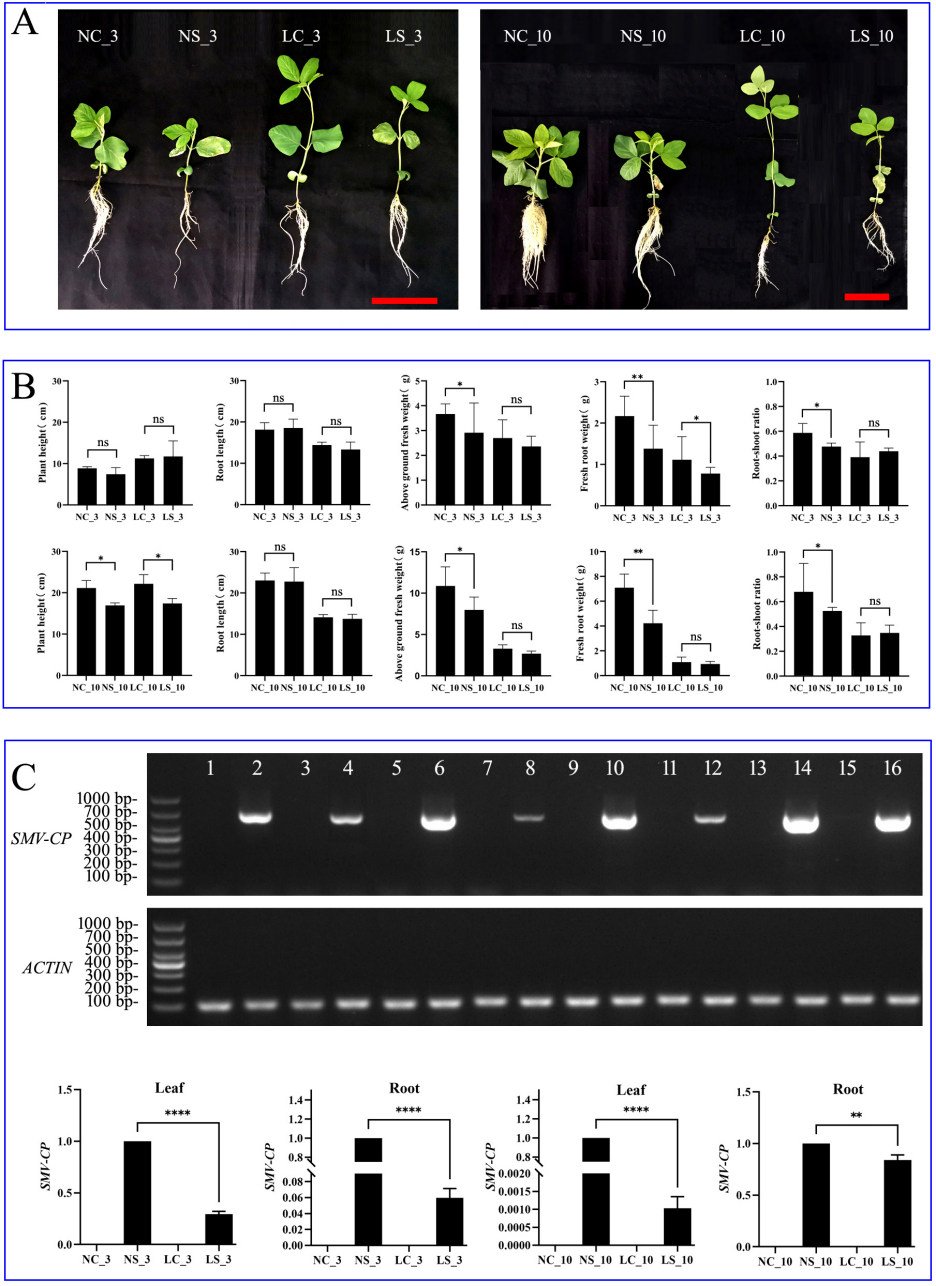
Morphology and viral replication levels in soybean plants under shaded and normal light conditions. **(A)** Growth status of soybean plants at 3 and 10 days post virus inoculation; **(B)** Determination of morphological parameters of soybean plants at 3 and 10 days post virus inoculation. NC_3, soybean plants under normal light with water treatment (healthy control) at 3 days; NS_3, soybean plants under normal light with virus inoculation at 3 days; LC_10, soybean plants under shaded conditions with water treatment (healthy control) at 10 days; LS_10, soybean plants under shaded conditions with virus inoculation at 10 days; **(C)** Viral replication in leaves and roots of soybean plants under shaded and normal light conditions. Lanes 1-4: Viral content detection in soybean leaves from NC, NS, LC, and LS treatment groups at 3 days post inoculation (dpi); Lanes 5-8: Viral content detection in soybean roots from NC, NS, LC, and LS treatment groups at 3 dpi; Lanes 9-12: Viral content detection in soybean leaves from NC, NS, LC, and LS treatment groups at 10 dpi; Lanes 13-16: Viral content detection in soybean roots from NC, NS, LC, and LS treatment groups at 10 dpi (Original gel images are provided in **Supplementary Figure S1** of Original Data). NC, healthy control group under normal light; NS, virus-inoculated group under normal light; LC, healthy control group under shaded conditions; LS, virus-inoculated group under shaded conditions. Scale bar = 10 cm. Data are presented as the means ± standard deviations. * indicates p<0.05, ** indicates p<0.01, **** indicates p<0.0001.

After the analysis of plant height, root length, root weight, shoot weight, and the root-to-shoot ratio in soybean, the results revealed significant impacts of shading and SMV infection on soybean growth and development (**Figure 1A**). On the third day post-SMV infection, no significant changes in the height or fresh shoot weight of the infected plants were detected. However, under shaded conditions with SMV inoculation, the root length and root weight significantly differed, with the root-to-shoot ratio markedly lower than that in the control group. Ten days following virus inoculation, variations in plant height, fresh shoot weight, root length, and root weight were noted among the different treatments. Under normal light conditions, the average plant height of the soybeans was 21.13 cm, the fresh shoot weight was 10.87 g, the root length was 23 cm, the root weight was 7.09 g, and the root-to-shoot ratio was 0.679. Conversely, after virus inoculation, the plant height decreased to 16.93 cm (a reduction of 4.2 cm), the fresh shoot weight decreased to 7.91 g, the root length decreased to 22.73 cm, and the root weight decreased significantly to 4.22 g (a reduction of 2.87 g), resulting in a root-to-shoot ratio of 0.525. Under shaded conditions, the average plant height was 22.17 cm, whereas the fresh shoot weight significantly decreased to 3.28 g (a decrease of 7.59 g compared with that of the control group). The root length was 14.1 cm (an 8.9 cm reduction), the root weight was 1.084 g (a decrease of 6 g), and the root-to-shoot ratio was 0.328. In contrast, under shaded and virus-inoculated conditions, the plant height was 17.4 cm, the fresh shoot weight was 2.677 g, the root length was 13.73 cm, the root weight decreased to 0.94 g, and the root-to-shoot ratio was 0.348 (**Figure 1B**).

On the third and tenth days after virus inoculation, the SMV content in the leaves and roots was measured. The results of RT–PCR revealed band sizes of approximately 670 bp (**Figure 1C**, **Supplementary Figure S1**), confirming the occurrence of SMV infection. The expression levels of the SMV coat protein-encoding gene (SMV-CP) in the leaves and roots of soybean plants were assessed via RT–qPCR. Three days postinoculation, the SMV content in the V0 leaves of soybean plants under shaded conditions was 70.6% lower than that in the V0 leaves of normally infected plants, while the SMV content in the roots decreased by 95%. By ten days postinoculation, the SMV content in the V1 leaves of shaded soybeans was dramatically lower (by 99%) than that in the V1 leaves of normally infected plants, whereas the SMV content in the roots was 16.1% lower than that in the control group (**Figure 1C**). These findings indicate that shaded conditions can significantly reduce the accumulation of SMV in both soybean leaves and roots. Elevated viral replication under normal light may result from defense-induced susceptibility, where ROS-hypersensitive responses compromise cellular integrity, facilitating infection. Conversely, viral suppression under shade reflects not absolute resistance but dynamic host-pathogen resource allocation. Preserved host fitness inherently constrains obligate biotrophic pathogens.

### 3.2 Shade and SMV infection promote phosphorus uptake in soybeans

**Figure 2:**
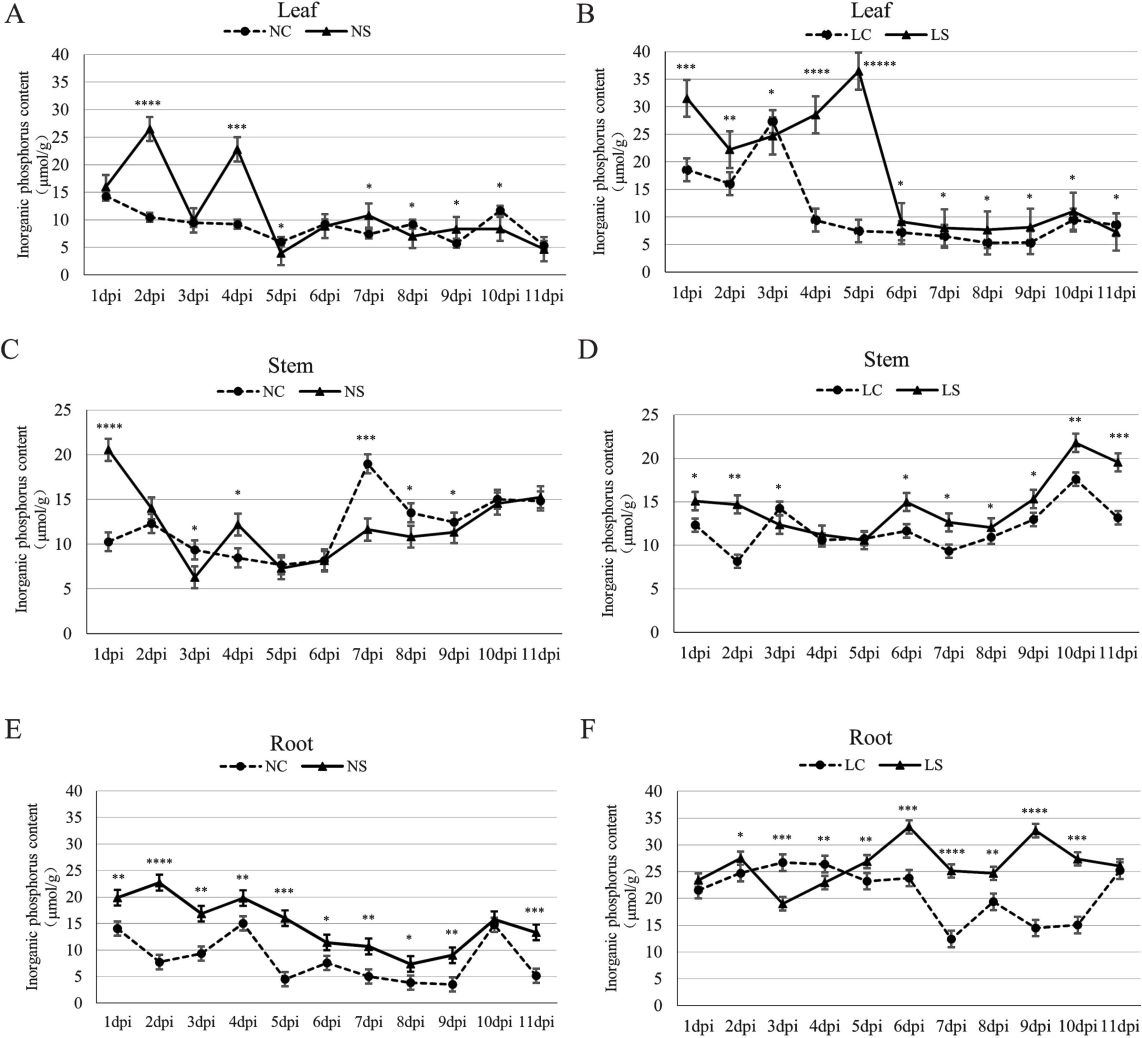
Dynamic changes in inorganic phosphorus content in soybean plants under normal light and shaded conditions. **(A, C, E)** show the changes in inorganic phosphorus content in leaves, stems, and roots, respectively, of non-inoculated (healthy) and virus-inoculated soybean plants under normal light conditions; **(B, D, F)** show the dynamic changes in inorganic phosphorus content in leaves, stems, and roots, respectively, of non-inoculated (healthy) and virus-inoculated soybean plants under shaded conditions. NC, healthy control group under normal light; NS, virus-inoculated group under normal light; LC, healthy control group under shaded conditions; LS, virus-inoculated group under shaded conditions. Data are presented as the means ± standard deviations. * indicates p<0.05, ** indicates p<0.01, *** indicates p<0.001, **** indicates p<0.0001, ***** indicates p<0.00001.

Under normal light conditions, inorganic phosphorus absorption and its translocation to leaves were significantly enhanced during the early stages of viral infection (days 1~6) (**Figures 2A, C, E**). This early accumulation of phosphorus in leaves may serve as a substrate for the synthesis of defense-related metabolites, which is consistent with the later-observed role of *miR399j* in directing phosphorus to leaves to enhance defense responses. In contrast, under shaded conditions, a distinct pattern was observed: inorganic phosphorus absorption and translocation efficiency were notably increased during the same early infection period (days 1~6) (**Figures 2B, D, F**). Importantly, when shading was combined with viral infection, the increased phosphorus absorption was predominantly attributed to the shading effect rather than the viral infection itself, suggesting that shade-induced stress may override the phosphorus allocation changes typically associated with viral infection.

Further tissue-specific analysis revealed that viral infection elicited significant dynamic changes in inorganic phosphorus levels in roots and leaves compared to uninfected controls, while phosphorus content in stems remained relatively unchanged (**Figure 2**). This tissue-specific variation suggests a systemic phosphorus reallocation strategy (potentially mediated through adjustments in root-to-shoot transport) that may be regulated by factors such as *miR399j* and its target gene. Collectively, these initial findings indicate that both light conditions (normal *vs*. shaded) and viral infection jointly influence inorganic phosphorus dynamics in a tissue- and time-dependent manner, providing a foundation for understanding the microRNA-mediated regulatory networks that govern phosphorus allocation, plant growth, and defense responses in soybean.

### 3.3 Effects of normal light and shade on defense gene expression

**Figure 3:**
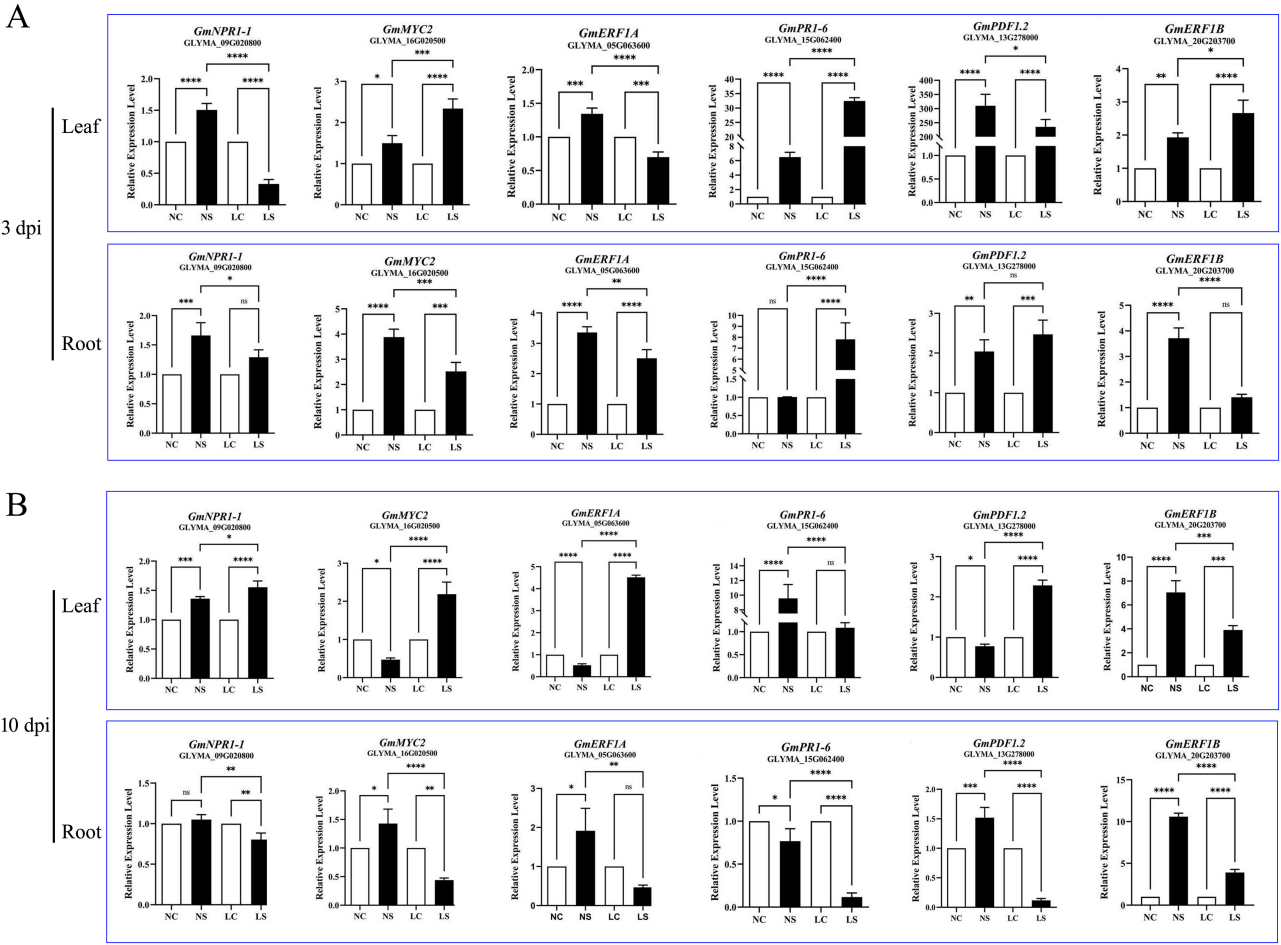
Expression profiling of defense-related genes in soybean leaf and root tissues following virus inoculation on the 3rd and 10th days after virus inoculation. **(A)** Detection of defense-related gene expression in soybean leaves and root tissues on the 3rd day postinoculation; **(B)** Detection of defense-related gene expression in soybean leaves and root tissues on the 10th day postinoculation. Data are presented as the means ± standard deviations. * indicates p<0.05, ** indicates p<0.01, *** indicates p<0.001, **** indicates p<0.0001.

To further analyze the effects of light environment changes on disease-susceptible soybean, we analyzed defense-related genes in SA and JA pathways. On the third day after infection with *Soybean mosaic virus* (SMV), the SA signaling pathway gene *GmNPR1–1* was significantly up-regulated in leaves under normal light conditions, indicating its key role in antiviral defense (**Figure 3A**). However, under shaded conditions, genes related to the SA signaling pathway were significantly suppressed. The expression of the downstream gene *GmPR1–6* increased, especially with greater up-regulation under shaded treatment. These findings suggest that shading may increase the expression of the *GmPR1–6* gene. The expression of the jasmonic acid (JA) signaling pathway gene *GmMYC2* increased after infection with SMV. Compared with that under normal light, the up-regulation of this gene under shaded conditions was greater. *GmPDF1.2* was induced by SMV infection under both light conditions, its absolute expression level remains higher under normal light than under shade. In terms of the ethylene (ET) signaling pathway, under normal light, the expression of the *GmERF1A* gene significantly increased after infection with SMV. However, under shaded conditions, the expression of *GmERF1A* was suppressed at 3 days post SMV infection, which may reflect a negative regulatory effect of shading on canonical ethylene (ET) signaling. In contrast, *GmERF1B* exhibited stronger up-regulation under shaded conditions compared to normal light, suggesting it is governed by distinct regulatory mechanisms. This differential response implies that *GmERF1B* may be activated through ET-independent pathways—potentially involving phytochrome-mediated light signaling or cross-talk with other hormonal networks—thereby enabling its induction even when overall ET sensitivity is reduced under shade. In root tissues, SMV infection triggered the up-regulation of the salicylic acid (SA) signaling marker gene *GmNPR1-1*. Notably, under shaded conditions, SMV infection led to a significant increase in the expression of *GmPR1-6*, another SA-responsive gene. The expression pattern of *GmMYC2*, a key regulator of jasmonic acid (JA) signaling, resembled that of *GmNPR1-1*, showing greater induction under normal light than under shade. Conversely, the JA/ET-responsive gene *GmPDF1.2* displayed stronger up-regulation under shaded conditions. Together, these findings highlight the complex interplay between light availability and hormone-mediated defense pathways during antiviral responses, with light playing a pivotal role in shaping the transcriptional landscape of soybean immunity.

Compared with that in the control group, on the 10th day after infection with SMV, the expression of the salicylic acid (SA) signaling pathway gene *GmNPR1–1* in leaf tissue was significantly up-regulated under both normal light and shaded conditions, especially under shaded conditions (**Figure 3B**). This may be related to the appropriate enhancement of defense in the late stage of viral infection in the shaded environment. Under normal light, the expression of the SA signaling pathway downstream gene *GmPR1–6* was still significantly increased on the 10th day after infection. Under shaded conditions, there was no significant difference in the gene expression of *GmPR1–6* on the 10th day after infection, while the expression of the *GmMYC2* and *GmPDF1.2* genes was significantly up-regulated, indicating that the shaded environment has an activating effect on the jasmonic acid (JA) signaling pathway in the late stage of SMV infection. In terms of the ethylene (ET) signaling pathway, under normal light, the expression of the *GmERF1A* gene was inhibited in the late stage of viral infection, whereas its expression was significantly increased under shaded conditions. Moreover, the expression of the *GmERF1B* gene was significantly up-regulated after SMV infection, and the increase under normal light conditions was greater than that under shaded conditions. Compared with that in the control group, the expression of the *GmNPR1–1* and *GmPR1–6* genes in the root tissue was downregulated under shaded conditions in the late stage of SMV infection, indicating that the antiviral defense role of the SA signaling pathway in the root tissue was weakened. In the late stage of infection under shaded conditions, the expression of the JA signaling pathway genes *GmMYC2* and *GmPDF1.2* was inhibited. In contrast, the expression of the *GmERF1A* and *GmERF1B* genes was up-regulated under normal light, indicating that light plays a role in regulating the ET signaling pathway in root tissue.

In summary, light conditions profoundly influence the defense responses of soybean during SMV infection. Under normal light, the salicylic acid (SA) and ethylene (ET) signaling pathways are more robustly activated, leading to stronger induction of associated defense genes. In contrast, shaded conditions attenuate the expression of certain canonical defense markers—likely due to modulation of SA/ET signaling and suppression of Reactive oxygen species (ROS) bursts. However, this does not necessarily translate into compromised antiviral outcomes; indeed, viral accumulation is lower under shade at both 3 and 10 dpi. This apparent paradox may be explained by the dual role of ROS in SMV infection (where excessive levels can favor viral spread) and by resource-allocation trade-offs that prioritize survival under low-light stress. Thus, the relationship between defense gene activation and actual antiviral efficacy is context-dependent and shaped by the integrated effects of light, hormone signaling, and redox homeostasis.n summary, light conditions have a significant effect on the defense mechanisms of soybeans during the SMV infection process. Normal light helps to activate the expression of genes related to the SA and ET signaling pathways, increasing the antiviral ability of plants. However, the shaded environment may inhibit the expression of defense-related genes by affecting the normal functions of these signaling pathways or other defense-related signaling pathways, reducing the antiviral ability of plants.

### 3.4 GO enrichment analysis and KEGG analysis of differentially expressed genes under different light conditions

**Figure 4:**
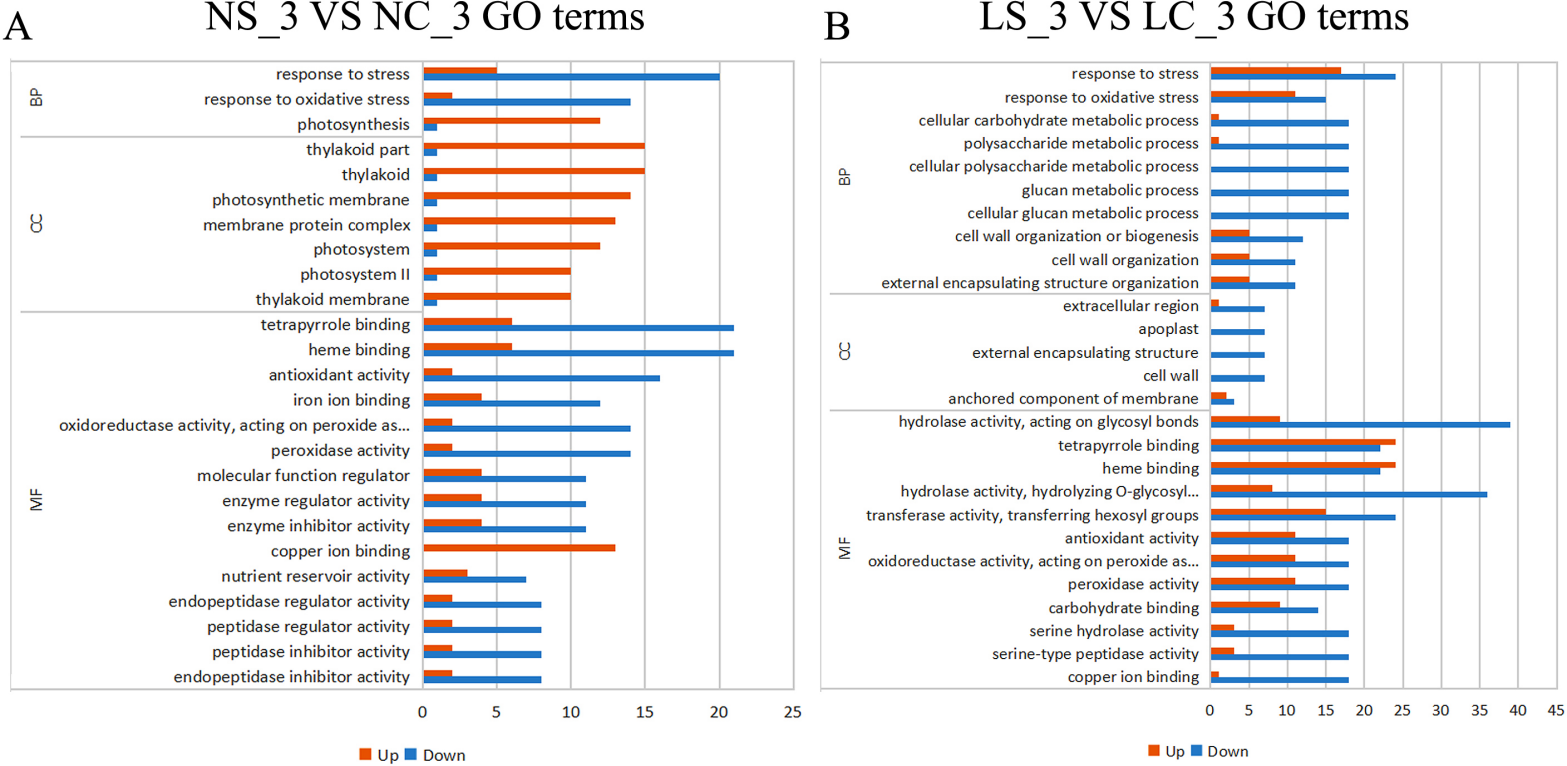
Gene Ontology (GO) enrichment analysis of differentially expressed genes under different treatments on day 3. **(A)** GO functional enrichment analysis of differentially expressed genes under normal light conditions on day 3; **(B)** GO functional enrichment analysis of differentially expressed genes under shaded treatment on day 3. MF (Molecular Function) refers to the molecular-level activities of gene products (e.g., catalysis, binding); BP (Biological Process) denotes the biological processes involving gene products (e.g., metabolism, signal transduction); CC (Cellular Component) describes the cellular location or environment of gene products.

**Figure 5:**
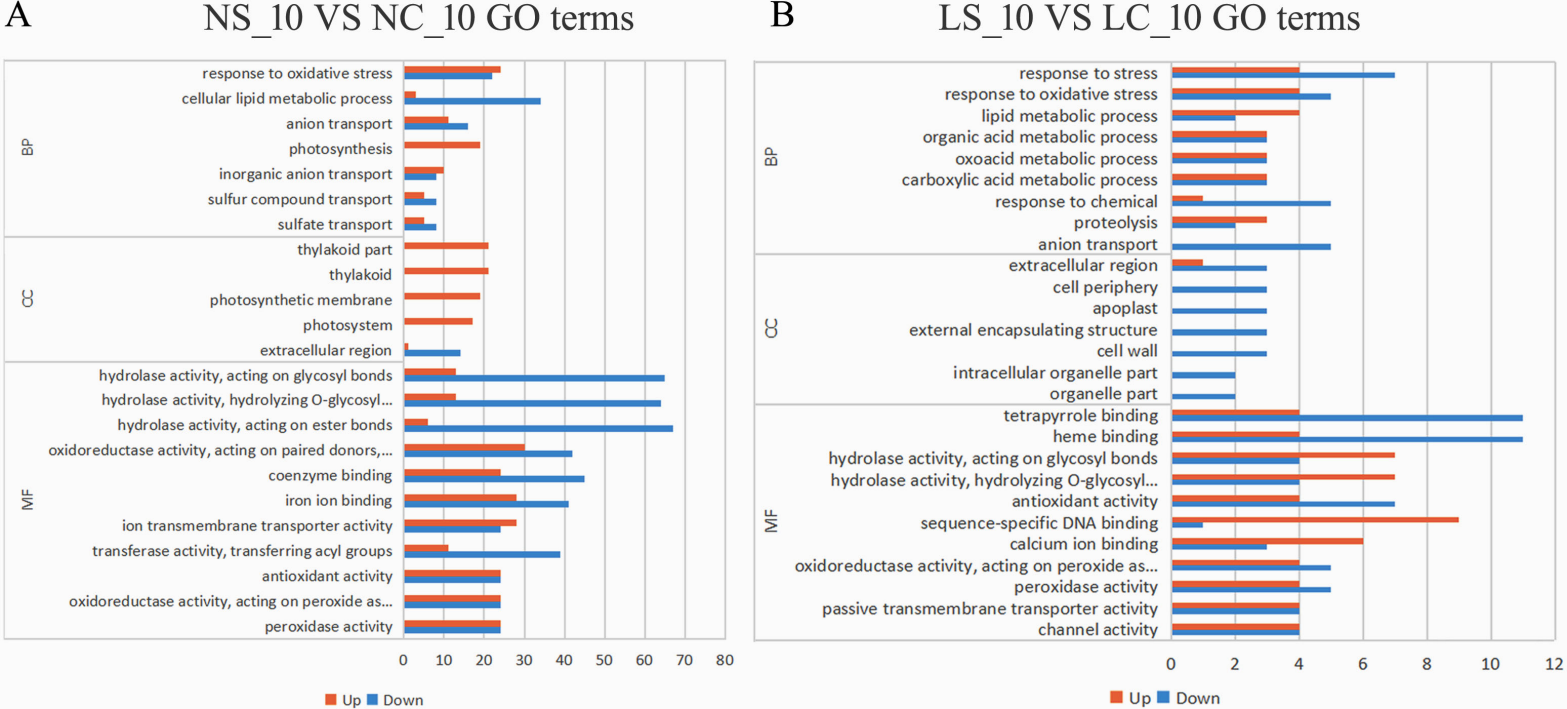
Gene Ontology (GO) enrichment analysis of differentially expressed genes under different treatments on day 10. **(A)** GO functional enrichment analysis of differentially expressed genes under normal light conditions on day 3; **(B)** GO functional enrichment analysis of differentially expressed genes under shaded treatment on day 10. MF (Molecular Function) refers to the molecular-level activities of gene products (e.g., catalysis, binding); BP (Biological Process) denotes the biological processes involving gene products (e.g., metabolism, signal transduction); CC (Cellular Component) describes the cellular location or environment of gene products.

At the same time, we performed GO enrichment analysis on the general transcriptome data. At Day 3 post-infection, infected plants prioritized growth adaptation over defense, marked by significant up-regulation of photosynthesis-related genes (e.g., photosynthesis, thylakoid, photosynthetic membrane) and suppression of stress responses (response to stress/oxidative stress). Molecular functions aligned with growth, such as copper ion binding up-regulation, while defense-related binding (e.g., heme/tetrapyrrole binding) was downregulated (**Figure 4**). By Day 10, a defense-oriented shift occurred: stress pathways (response to oxidative stress, cellular lipid metabolism, anion transport) were enriched, with photosynthesis genes remaining up-regulated. Crucially, extracellular defense structures (cell wall, apoplast) were uniformly downregulated, indicating compromised physical barriers. Molecular functions reflected enhanced transport (ion transmembrane transporter activity, nutrient reservoir activity) but widespread down-regulation of defense-linked activities (e.g., hydrolase/oxidoreductase activity, iron/antioxidant binding), highlighting a trade-off between sustained metabolic support and eroded structural/molecular defenses (**Figure 5**). Early infection favors growth and photosynthetic efficiency, while later stages intensify oxidative stress responses but fail to maintain critical cell wall/apoplast defenses, shifting molecular resources toward ion/nutrient transport.

**Figure 6:**
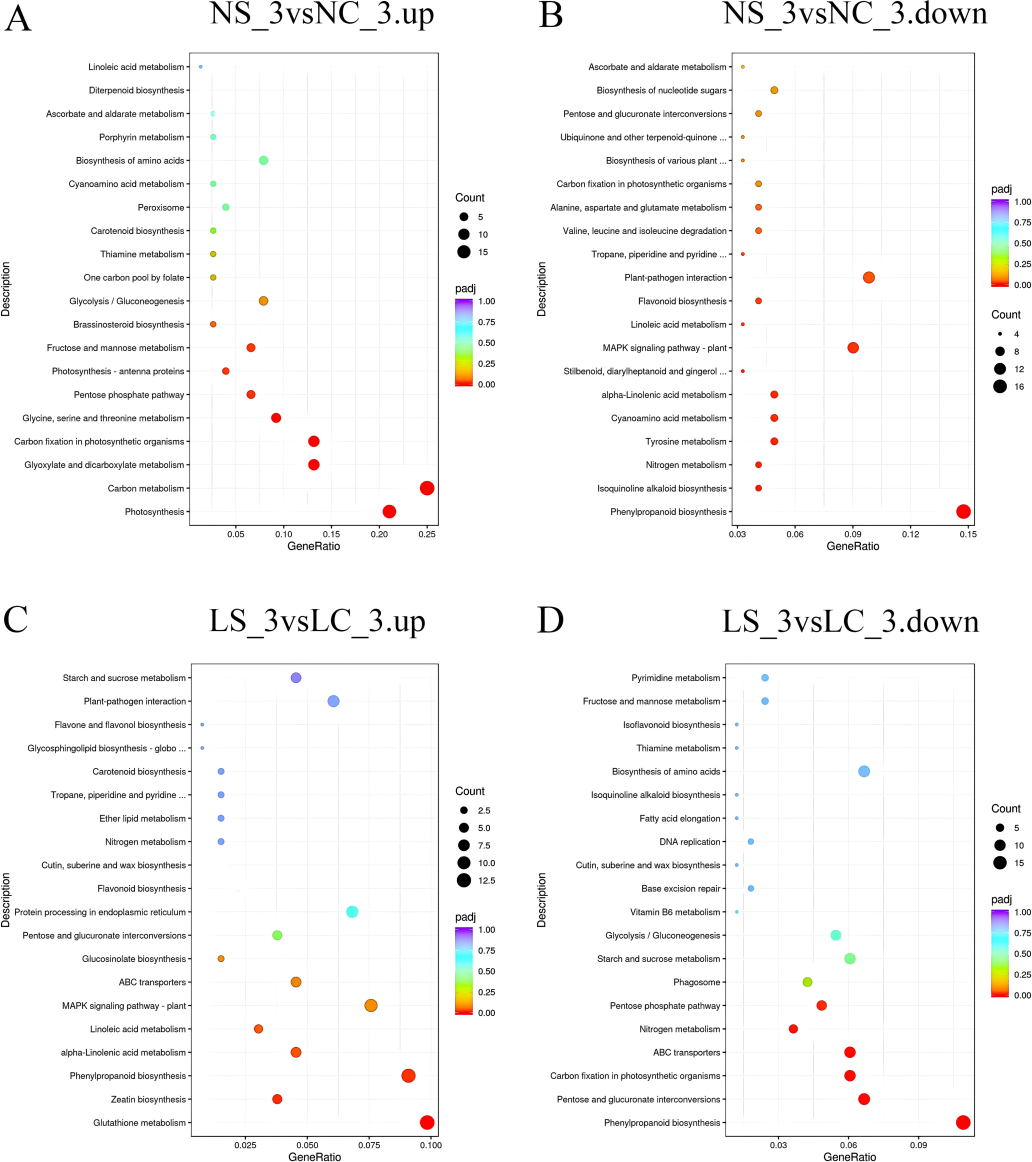
KEGG pathway enrichment of differentially expressed genes on day 3 **(A, B)** KEGG pathway enrichment analysis of up-regulated and down-regulated differentially expressed genes under normal light conditions, respectively; **(C, D)**, KEGG pathway enrichment analysis of up-regulated and down-regulated differentially expressed genes under shaded conditions, respectively. NC_3, soybean plants under normal light with water treatment (healthy control) at 3 days; NS_3, soybean plants under normal light with virus inoculation at 3 days; LC_10, soybean plants under shaded conditions with water treatment (healthy control) at 10 days; LS_10, soybean plants under shaded conditions with virus inoculation at 10 days.

**Figure 7:**
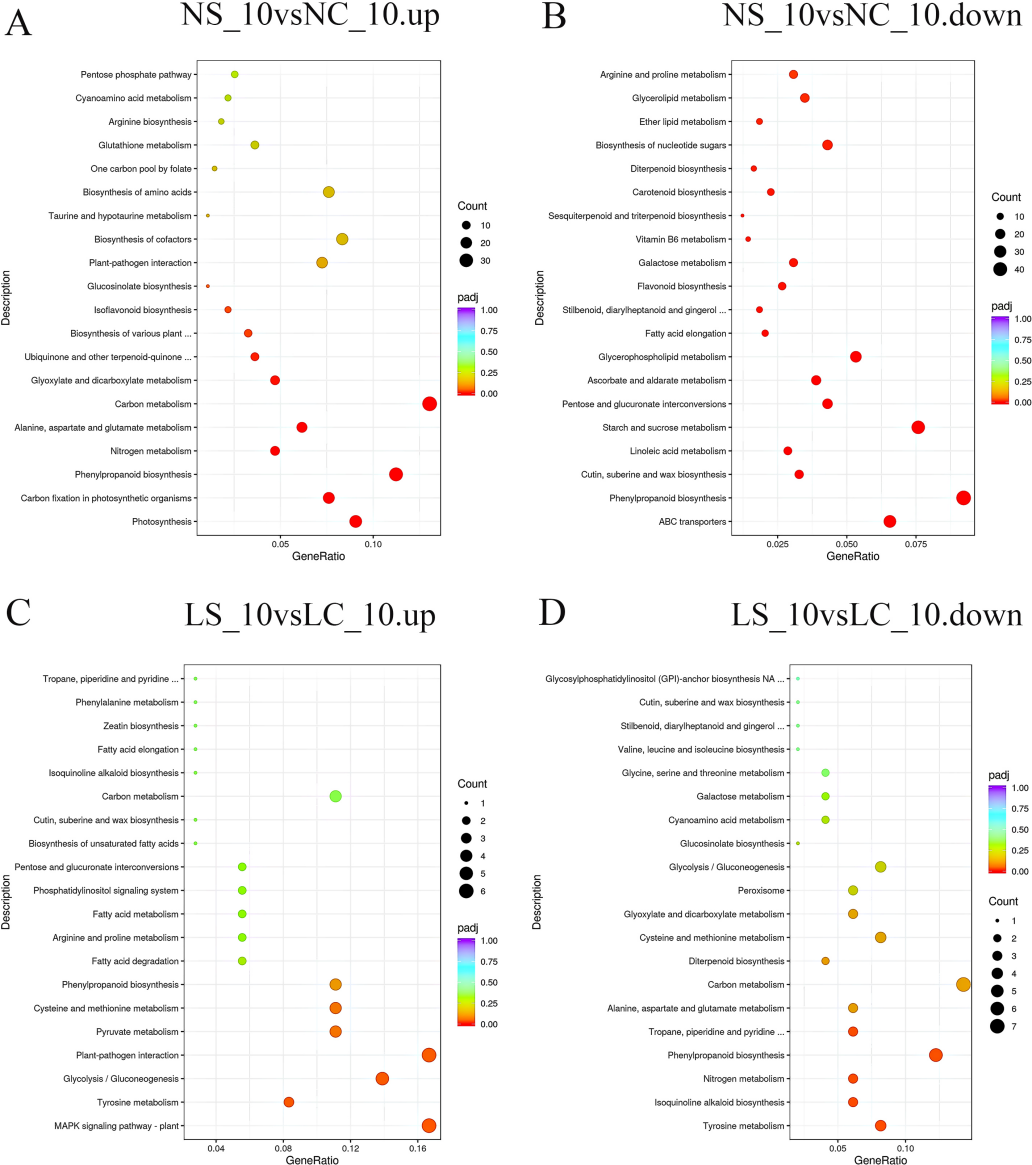
KEGG pathway enrichment of differentially expressed genes on day 10. **(A, B)** KEGG pathway enrichment analysis of up-regulated and down-regulated differentially expressed genes under normal light conditions, respectively; **(C, D)** KEGG pathway enrichment analysis of up-regulated and down-regulated differentially expressed genes under shaded conditions, respectively. NC_3, soybean plants under normal light with water treatment (healthy control) at 3 days; NS_3, soybean plants under normal light with virus inoculation at 3 days; LC_10, soybean plants under shaded conditions with water treatment (healthy control) at 10 days; LS_10, soybean plants under shaded conditions with virus inoculation at 10 days.

KEGG enrichment analysis revealed profound light-mediated restructuring of metabolic pathways in SMV-infected soybean roots. Under normal light at day 3 (**Figure 6**), SMV infection strongly induced carbon metabolism and photosynthesis but suppressed defense-related pathways (phenylpropanoid biosynthesis, plant-pathogen interaction, MAPK signaling). Conversely, under shade at day 3, SMV triggered induction of stress-mitigation pathways (glutathione metabolism, α-linolenic acid metabolism, zeatin biosynthesis) while suppressing diverse metabolic functions including phenylpropanoid biosynthesis, carbon fixation, ABC transporters, and nitrogen metabolism. By day 10 under normal light, SMV activated photosynthesis, carbon metabolism, and notably phenylpropanoid biosynthesis, while inhibiting starch/sucrose metabolism and glycerophospholipid metabolism. Strikingly, under shade at day 10 (**Figure 7**), SMV instead induced defense signaling (plant-pathogen interaction, MAPK signaling) and energy-generating pathways (glycolysis/gluconeogenesis), while repressing phenylpropanoid biosynthesis, tyrosine metabolism, and nitrogen metabolism. Critical light contrasts include: 1) Opposite regulation of phenylpropanoid biosynthesis (induced in normal light *vs*. suppressed in shade at D10), 2) Divergent defense signaling (suppressed in normal light at D3 but induced in shade at D10), and 3) Fundamentally altered energy strategies (photosynthesis/carbon fixation promoted only in normal light, while shade redirected energy flux toward glycolysis/gluconeogenesis and specialized stress metabolites).

**Figure 8:**
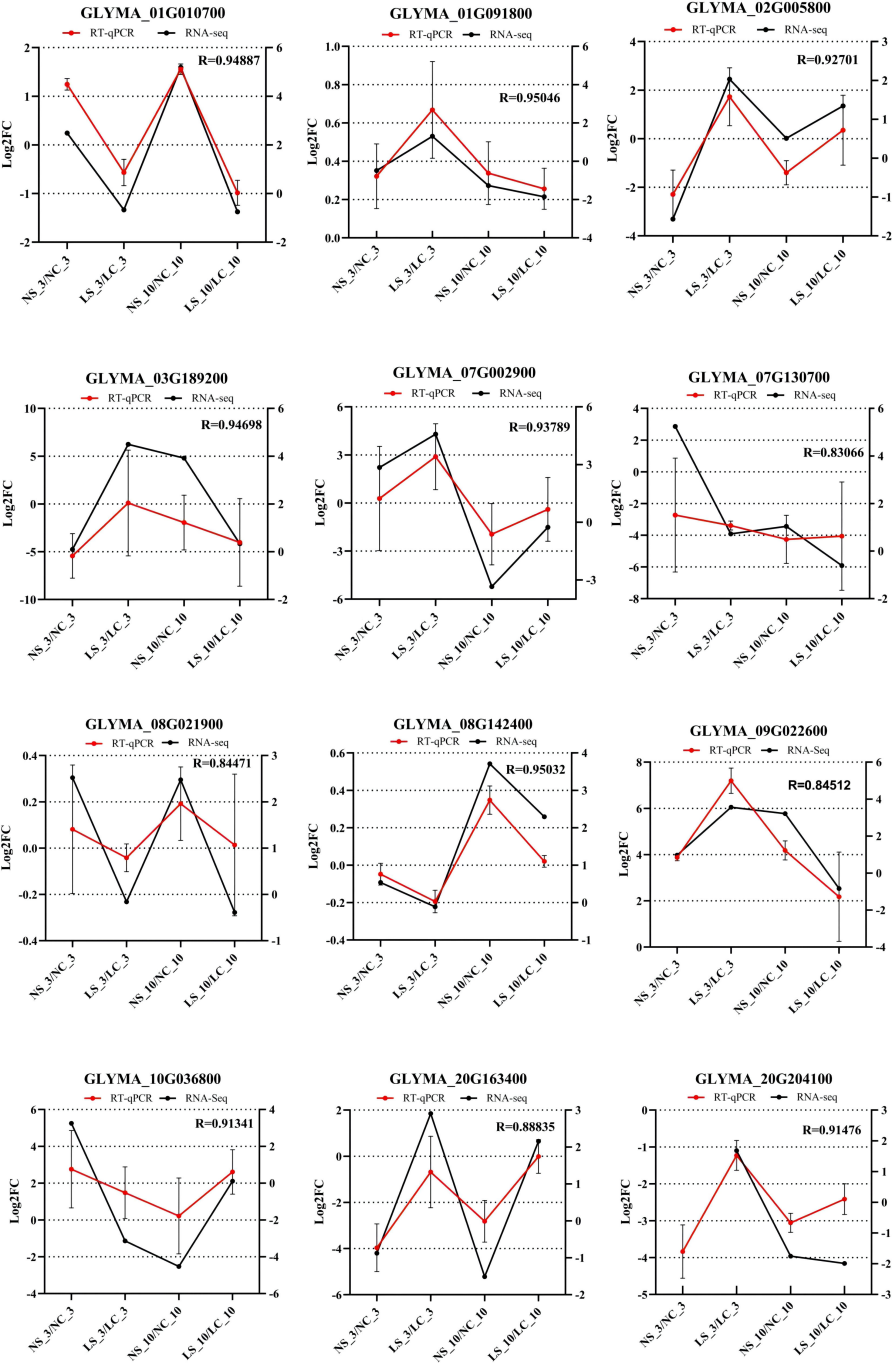
RT-qPCR and RNA-seq alignment analysis of 12 differentially expressed genes. NC_3, soybean plants under normal light with water treatment (healthy control) at 3 days; NS_3, soybean plants under normal light with virus inoculation at 3 days; LC_10, soybean plants under shaded conditions with water treatment (healthy control) at 10 days; LS_10, soybean plants under shaded conditions with virus inoculation at 10 days.

We conducted RT-PCR analysis on the differentially expressed genes in the transcriptome and found that the results were consistent with the quantitative results and the results of transcriptome sequencing. This proves the reliability of the transcriptome data (**Figure 8**).

### 3.5 Analysis and validation of differentially expressed miRNAs

**Figure 9:**
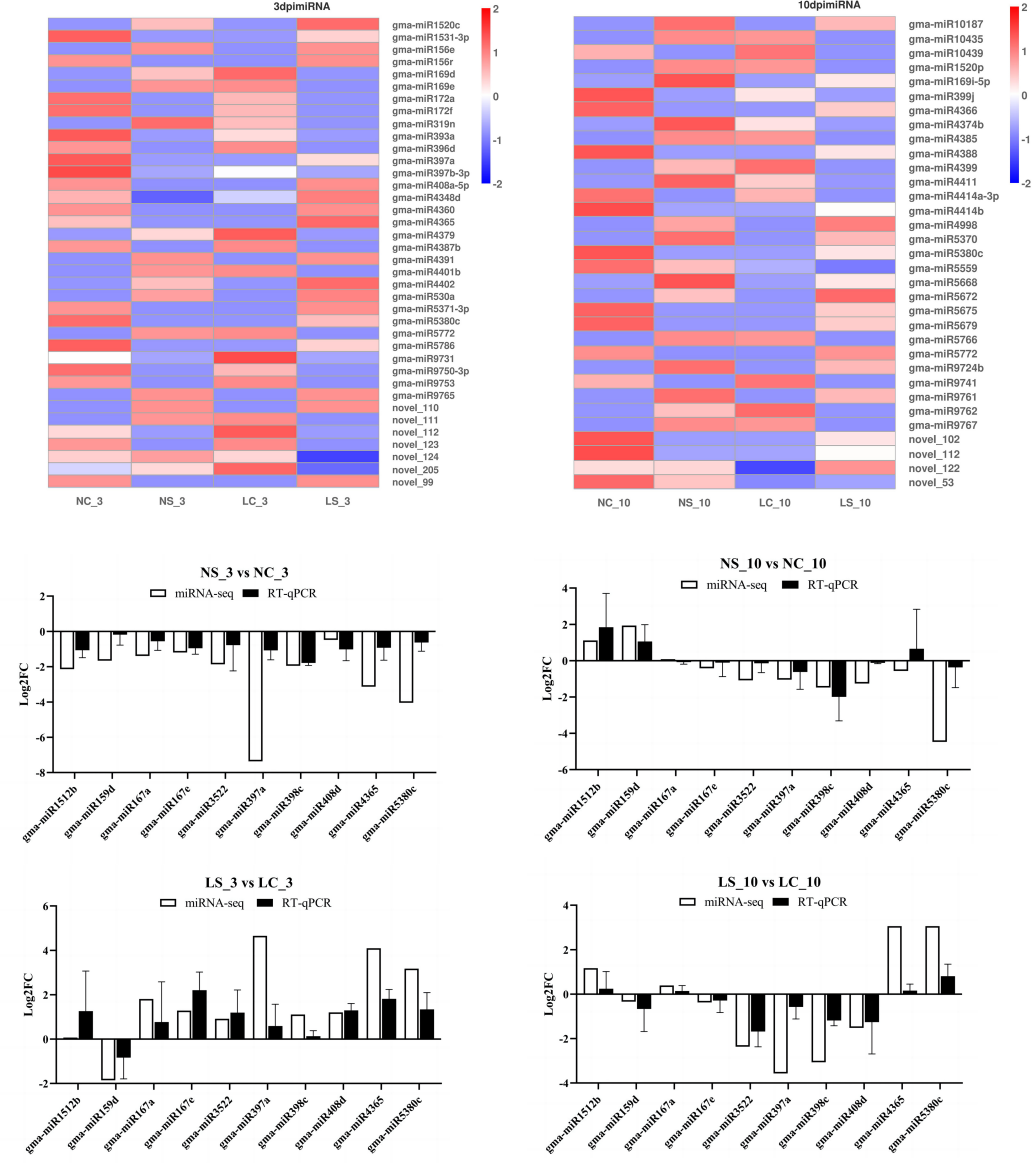
Analysis of differentially expressed miRNAs under normal light versus shade conditions and validation of resistance-related highly differentially expressed genes by RT–qPCR and transcriptome sequencing. NC_3 indicates Normal light + mock inoculation (water), on the third day; NS_3 indicates Normal light + virus inoculation on the third day; LC_10 indicates Shade + mock inoculation (water) on the tenth day; LS_10 indicates Shade + virus inoculation on the tenth day. Clustering was performed using log_10_ (TPM + 1) transformed expression values. In the heatmap, red indicates up-regulated miRNAs and blue indicates downregulated miRNAs relative to respective controls; color intensity corresponds to expression magnitude.

Using the expression data of miRNAs, we screened out miRNAs with a fold change of more than 3 on days 3 and 10 and performed a cluster analysis on the relationships between samples and miRNAs (**Figure 9**). The results revealed that viral infection caused significant changes in the expression levels of some miRNAs. Moreover, under both normal light and shaded conditions, the same miRNA significantly differed between the treatment group and the control group. We selected 10 miRNAs whose expression significantly differed on days 3 and 10 for RT–qPCR detection to verify the accuracy of the small RNA sequencing results. Although there were differences in the relative expression levels of the same miRNA under different conditions, the miRNA expression trends shown by the two quantitative methods were consistent, which confirmed the repeatability and reliability of the miRNA sequencing data.

### 3.6 Prediction of miRNAs and their target genes related to nutrient absorption and defense pathways

To gain deeper insight into the roles of miRNAs in soybean antiviral defense, we prioritized candidates associated with either pathogen response or low-phosphorus (Pi) stress and predicted their target genes (**Table 1**). Integrating small RNA-seq and mRNA-seq datasets under the principle of miRNA–mRNA negative correlation, we identified 108 high-confidence miRNA–mRNA regulatory pairs. Target prediction was performed using three complementary algorithms—psRNATarget, RNAhybrid, and psRobot—and further refined through integration of expression profiles, functional annotations, and literature evidence.

**Table 1:** miRNAs with significant differences and their target genes.

miRNA id	NS_3/NC_3	LS_3/LC_3	NS_10/NC_10	LS_10/LC_10	Target gene id	NS_3/NC_3	LS_3/LC_3	NS_10/NC_10	LS_10/LC_10	Description
	log2FC					log2FC				
gma-miR156e	3.21	3.18	0	4.52	GLYMA_03G117600	NA	-3.911	-5.695	-4.715	LRR receptor kinase-like protein
gma-miR167l	-1.39	1.81	0.08	0.39	GLYMA_13G221400	1.227	-1.638	-1.264	0.779	auxin response factor 6
gma-miR169d	3.21	-4.08	5.82	-0.25	GLYMA_16G005500	0.959	-1.675	-2.484	0.773	nuclear transcription factor Y subunit A-18
gma-miR169e	4.13	-4.08	5.56	-0.25	GLYMA_16G005500	0.959	-1.675	-2.484	0.773	nuclear transcription factor Y subunit A-18
gma-miR169j-5p	1.46	4.1	2.31	-3.29	GLYMA_08G335900	0.054	0.026	0.102	0.260	probable WRKY transcription factor 40
gma-miR397a	-7.36	4.65	-1.05	-3.57	GLYMA_01G108200	0.636	-0.160	3.257	0.115	laccase-7
					GLYMA_11G233400	2.503	-4.008	-1.383	0.060	laccase-12
gma-miR398c	-1.95	1.11	-1.48	-3.07	GLYMA_05G055000	-0.045	0.942	0.885	0.186	Cu/Zn-superoxide dismutase copper chaperone
gma-miR399a	1.06	-0.77	-3.2	-0.35	GLYMA_10G036800	5.247	-1.138	-2.528	2.112	inorganic phosphate transporter 1-4
gma-miR399j	3.21	0	-5.18	-3.29	GLYMA_14G188000	1.655	1.849	0.985	-0.141	inorganic phosphate transporter 1-11
					GLYMA_10G036800	5.247	-1.138	-2.528	2.112	inorganic phosphate transporter 1-4
gma-miR408d	-0.48	1.2	-1.26	-1.52	GLYMA_13G006700	-0.189	0.597	1.217	0.638	nudix hydrolase 2
gma-miR4348d	-4.19	3.21	2.62	-1.72	GLYMA_09G204500	-0.189	0.170	-0.295	-0.189	transcription factor MYC1
gma-miR4413b	-1.39	3.18	4.84	-0.81	GLYMA_03G208800	3.981	1.849	2.031	NA	auxin response factor 23
gma-miR530a	4.69	5.05	1.27	3.97	GLYMA_02G013900	2.223	4.292	5.768	-1.051	transcription factor MYB12
gma-miR5380c	-4.05	3.18	-4.47	3.06	GLYMA_06G178900	1.071	-0.586	-1.006	1.830	LBD domain-containing transcription factor
gma-miR5776	0	-3.16	5.56	-1.05	GLYMA_05G224300	3.981	3.042	NA	3.408	Myb_DNA-binding

Among these, four key interactions were selected for experimental validation: *miR397a* targeting *GmLAC7* (GLYMA_01G108200) and *GmLAC12* (GLYMA_11G233400), which encode laccases essential for lignin polymerization and cell wall fortification; *miR399j* targeting *GmPHT1-*4 (GLYMA_10G036800), a high-affinity Pi transporter central to Pi uptake and redistribution; and miR408d targeting *GmNUDT2* (GLYMA_13G006700), a Nudix hydrolase potentially involved in nucleotide homeostasis during stress signaling.

Notably, the expression dynamics of these miRNAs were modulated by both light environment and SMV infection. Under normal light, SMV triggered down-regulation of *miR397a*, likely derepressing laccase expression to enhance structural defense via lignin deposition. In contrast, shade induced *miR397a* up-regulation, potentially diverting resources from defense toward growth—a trade-off reflected in altered viral accumulation. Similarly, *miR399j* exhibited tissue- and time-dependent responses to SMV and light, suggesting its role in reprogramming Pi allocation to balance immunity and nutrient economy. Collectively, these targets may directly mediate antiviral responses or indirectly bolster resistance by modulating phosphorus acquisition, transport, and metabolic utilization, thereby linking nutritional status to immune output in a light-dependent manner.

### 3.7 Validation of the interaction between miRNAs and target genes

**Figure 10:**
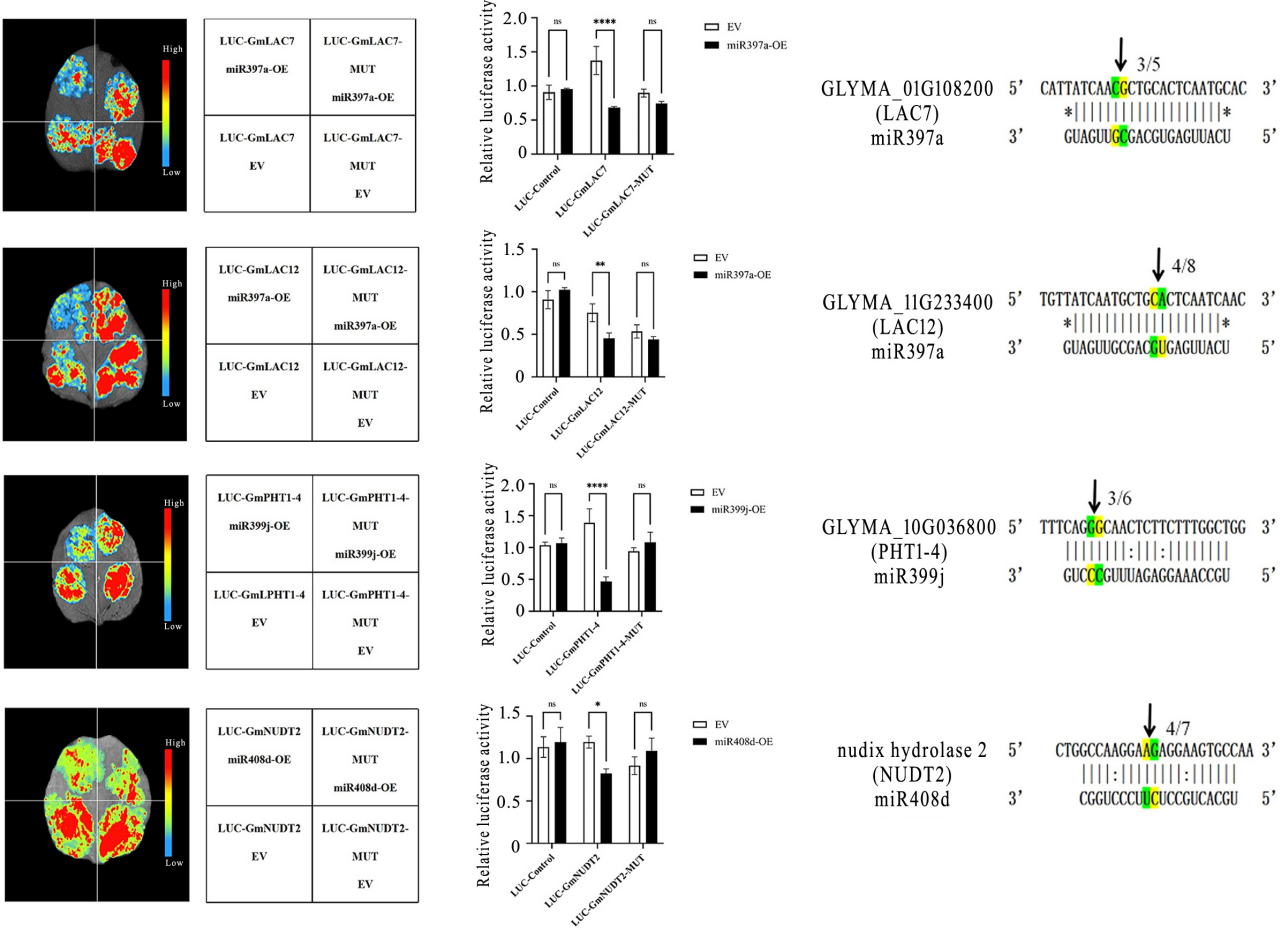
Validation of the targeting relationships between miRNAs and target genes through dual-luciferase assays and 5’ RACE experiments. Validation of the targeting relationship through dual-luciferase assays and 5’RACE experiments; in the dual-luciferase assay, LUC-Control represents the pGreenII 0800 empty vector; LUC-GmLAC7, LUC-GmLAC12, LUC-GmPHT1-4, and LUC-GmNUD2 represent the recombinant wild-type vectors of the target genes; LUC-GmLAC7-MUT, LUC-GmLAC12-MUT, LUC-GmPHT1-4-MUT, and LUC-GmNUD2-MUT represent the recombinant mutant vectors of the target genes; EV represents the pCAMBIA1300 empty vector; miRNA-OE represents the miRNA overexpression vector; * indicates p<0.05, ** indicates p<0.01, **** indicates p<0.0001; in the 5’RLM-RACE sequencing analysis and comparison results, each pair of lines represents a group, the first line is the ID and gene sequence of the target gene, and the second line is the sequence of the bound reverse miRNA. Complementary bases are indicated by vertical lines, G: U pairs are indicated by colons, and mismatches are indicated by *. The arrows in each group point to the mRNA cleavage site, and the numbers represent the frequency of sequencing clones.

The target gene mutant vector was cotransfected with miR397a-OE, miR399j-OE, or miR408d-OE into tobacco leaf cells for transient expression, and changes in fluorescence intensity were observed. The results revealed that when the wild-type target gene was coexpressed with miRNA-OE, the fluorescence intensity significantly decreased, indicating that there was a targeted regulatory relationship between the target gene and the miRNA (**Figure 10A**, left column). The dual-luciferase activity assay further confirmed the above conclusion, with a significant decrease in the ratio of firefly luciferase activity to Renilla luciferase activity (LUC/REN) (**Figure 10A**, middle column). The interactions of soybean *miR397a*, *miR399j*, and *miR408d* with *GmLAC7, GmLAC12, GmPHT1-4*, and *GmNUD2* interfere with the expression of the target gene at the protein level, hindering the translation of the firefly luciferase protein, which in turn reduces enzyme activity. The above results collectively demonstrate that *GmLAC7* and *GmLAC12* are target genes of *miR397a*, *GmPHT1–4* is a target gene of *miR399j*, and *GmNUD2* is a target gene of *miR408d*.

To verify the cleavage effect of miRNA on specific sequences of the target gene, this study employed the 5’RLM-RACE experimental technique to detect the interaction between the four pairs of miRNAs and their potential target genes (**Figure 10A**, right column). Sequence alignment analysis revealed that the sequencing sequence was highly consistent with the expected cleavage site sequence of the miRNA target gene, confirming that the mRNAs of these four target genes were indeed cleaved at the target sites of the corresponding miRNAs. **Supplementary Figure S2A** (Supplementary Material, original gel images are showed in **Supplementary Figure S3**) shows the results of PCR detection of the *GmLAC7, GmLAC12, GmPHT1-4*, and *GmNUDT2* genes. **Supplementary Figure S2B** (Supplementary Material, original gel images are showed in **Supplementary Figure S3**) presents the results of colony PCR detection for the target genes mentioned above.

### 3.8 Construction of *miR397a* and *miR399j* overexpression and silencing lines

To investigate the functional significance of the target miRNA, we performed mutant construction experiments. Specific primers designed on the basis of the miRNA precursor gene sequence (**Supplementary Figure S4A**; **Supplementary Table S2**, original gel images are showed in **Supplementary Figure S5**) were used with soybean root cDNA as a template, and we successfully performed PCR amplification, resulting in an amplified fragment of approximately 400 bp (**Supplementary Figures S4B, C**, original gel images are showed in **Supplementary Figure S5**). The target fragment was recovered via 1% agarose gel electrophoresis, and the plasmid vector was subjected to double digestion, followed by homologous recombination with the target fragment. The recombinant plasmid was transformed into *E. coli*, and the bacterial mixture was subjected to PCR electrophoresis, which revealed clear bands. The sequencing results were consistent with no errors, and the plasmid was ultimately transformed into Agrobacterium. On the basis of the size of the electrophoresis bands and sequencing results, we preliminarily determined that the recombinant overexpression vector was successfully constructed.

Using the linker sequence linker-STTM (**Supplementary Figures S4D, E**, original gel images are showed in **Supplementary Figure S5**), the STTM-miR397a and STTM-miR399j fragments were amplified via PCR, recovered, and inserted into the intermediate vector. Colony PCR electrophoresis (**Supplementary Figures S4F, G**, original gel images are showed in **Supplementary Figure S5**) and sequencing verification confirmed that the pEasy-STTM-397a and pEasy-STTM-399j lines were successfully constructed. The obtained STTM sequence-containing clone vector and vector were subsequently subjected to double digestion, ligated with T4 ligase, and transformed.

On the third day after irrigation, we extracted the leaf and root tissues of the mutant soybean plants and detected the expression levels of *miR397a* and *miR399j* (**Supplementary Figures S6A–D**). Compared with those in the control plants, the expression levels of *miR397a* in the leaf and root tissues increased by 257.4% and 79.5%, respectively. The expression levels of *miR399j* in the leaf and root tissues were 101% and 34.9% greater than those in the control group, respectively. Conversely, in the silenced plants, the expression levels of *miR397a* in the leaf and root tissues decreased by 27.6% and 61.2%, respectively, compared with those in the control group; the expression levels of *miR399j* in the leaf and root tissues decreased by 50.1% and 35.3%, respectively, compared with those in the control group. Influenced by multiple factors, the efficiency of the overexpression and silencing of different miRNAs differs.

### 3.9 SMV accumulation in *miR397a*- and *miR399j*-modified lines under contrasting light regimes

**Figure 11:**
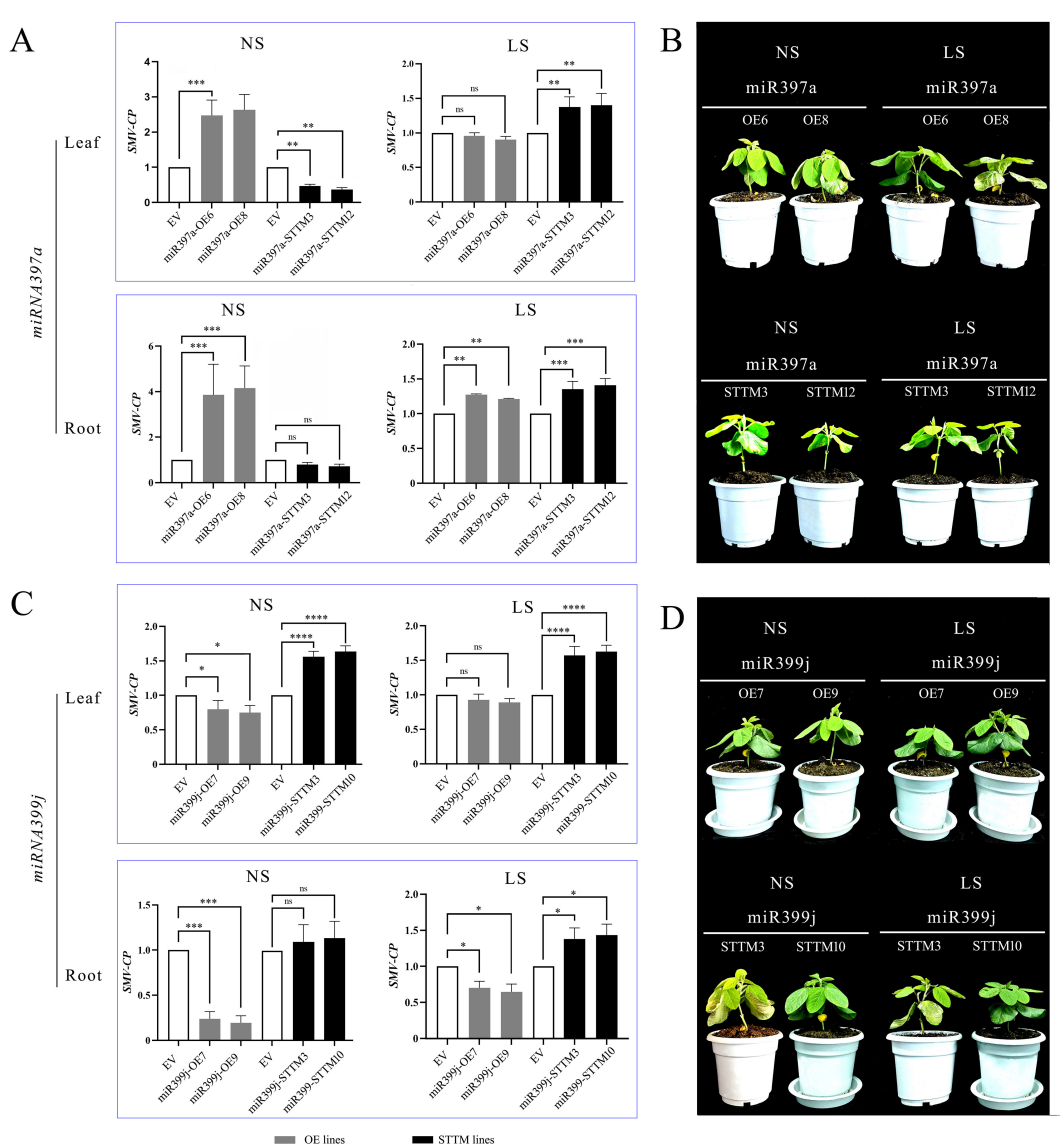
Analysis of viral accumulation and phenotype in miRNA-silenced lines. **(A)** Viral accumulation in leaf and root tissues of the *miR397a*-silenced mutant.; **(B)** Representative phenotype of the *miR397a*-silenced mutant. **(C)** Viral accumulation in leaf and root tissues of the *miR399j*-silenced mutant; **(D)** Representative phenotype of the *miR399j*-silenced mutant. NS represents the treatment group inoculated with virus under normal light; LS represents the treatment group inoculated with virus under shaded conditions. The empty vector (EV) control plants are represented by bars positioned to the right of the mutant bars for visual comparison (set as 1). Data are presented as the means ± standard deviations. * indicates p<0.05, ** indicates p<0.01, *** indicates p<0.001, **** indicates p<0.0001.

Comprehensive analysis of SMV accumulation in *miR397a*- and *miR399j*-modified lines under contrasting light regimes (**Figure 11**) revealed a critical light-dependent interplay between these miRNAs and pathogen defense. Under normal light conditions, *miR397a* functioned as a negative regulator of resistance: SMV content significantly increased in leaves and roots of miR397a-overexpressing (OE) lines but decreased in miR397a-silenced lines. Conversely, *miR399j* acted as a positive regulator of resistance: SMV accumulation increased in miR399j-silenced plants but decreased in miR399j-OE lines. This demonstrates an antagonistic relationship between these miRNAs in modulating basal defense against SMV under optimal light. Strikingly, shade stress overrode these specific miRNA-mediated regulatory pathways, resulting in a universal increase in SMV content across all genotypes – including both OE and silenced lines for both miRNAs – in both leaves and roots. This phenotypic reversal indicates that the low-light environment fundamentally compromises host resistance capacity, irrespective of the manipulated miRNA’s inherent function under normal light. Collectively, these results provide direct phenotypic evidence that shade stress disrupts the finely tuned balance between miRNA-regulated processes and pathogen defense, favoring susceptibility.

### 3.10 *miR397a* negatively regulates *GmLAC7* and *GmLAC12*, compromising defense

**Figure 12:**
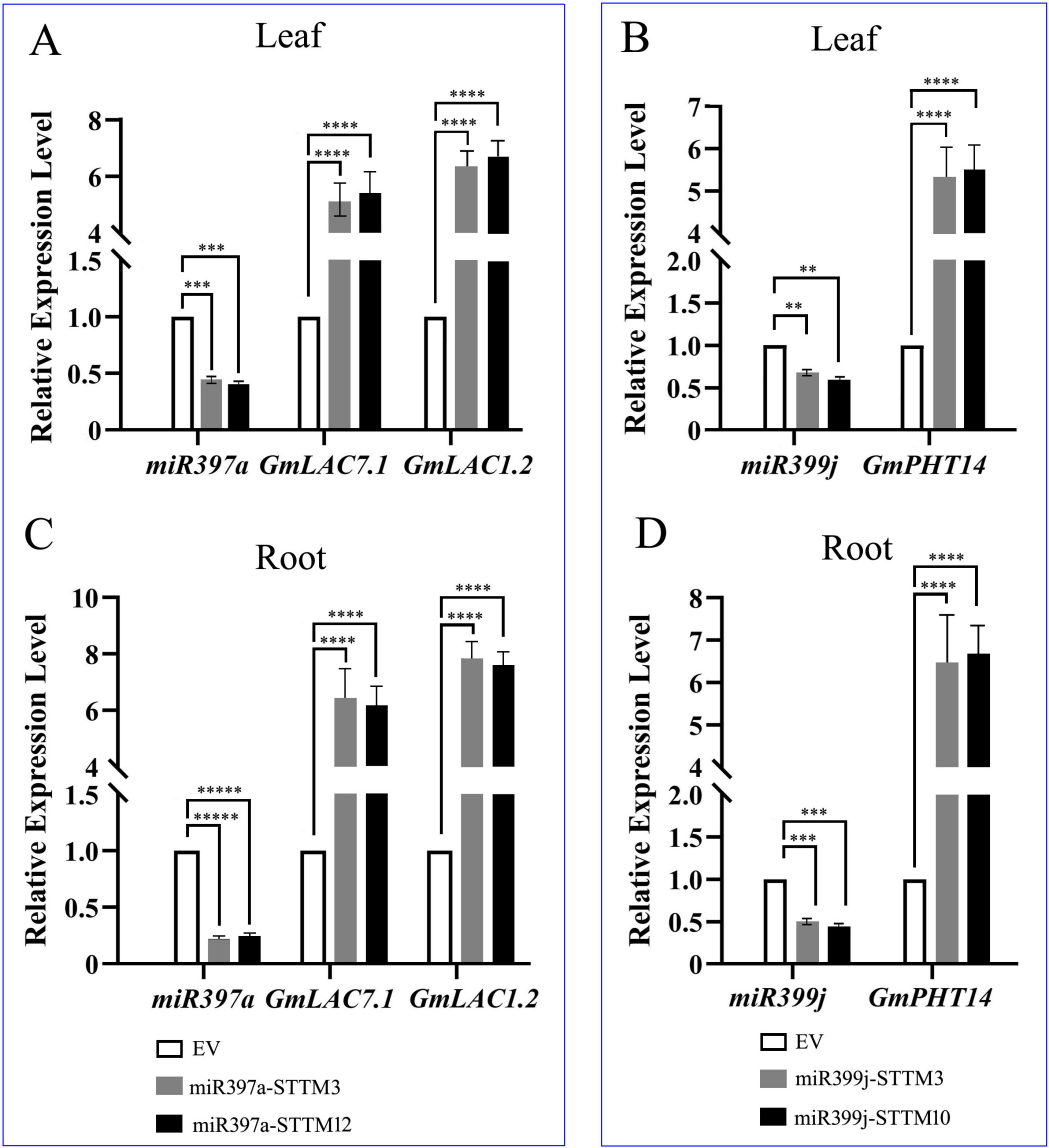
Expression analysis of mutant miRNAs and their target genes. **(A)** Expression levels of *miR397a*, *GmLAC7*, and *GmLAC12* in leaf tissue of the *miR397a*-silenced mutant; **(B)** Expression levels of *miR399j* and *GmPHT1–4* in leaf tissue of the *miR399j*-silenced mutant; **(C)** Expression levels of *miR397a, GmLAC7*, and *GmLAC12* in root tissue of the *miR397a*-silenced mutant; **(D)** Expression levels of *miR399j* and *GmPHT1–4* in root tissue of the miR399j-silenced mutant. EV, Empty vector control plants for the miR397a or miR399j-STTM line. The empty vector (EV) control plants are represented by bars positioned to the right of the mutant bars for visual comparison (set as 1). Data are presented as the means ± standard deviations. ** indicates p<0.01, *** indicates p<0.001, **** indicates p<0.0001, ***** indicates p<0.00001.

To elucidate the mechanism underlying miR397a-mediated susceptibility observed in **Figure 11A**, we analyzed its target laccase genes [*GmLAC7* (GLYMA_01G108200) and *GmLAC12* (GLYMA_11G233400)] in roots under normal light. *miR397a* silencing enhanced *GmLAC7/12* expression in leaf and root (**Figures 12A, C**). Given that laccases catalyze lignin polymerization (a critical component of physical defense barriers) this inverse correlation explains the increased SMV susceptibility in miR397a-OE lines (**Figure 11A**). Thus, *miR397a* promotes viral vulnerability by repressing lignin-biosynthetic genes, weakening structural defense mechanisms.

Shade stress overrode these specific miRNA-mediated regulatory pathways, resulting in a universal increase in SMV content across all genotypes in both leaves and roots (**Figure 11**). Notably, this compromised resistance extended to miR397a-silenced lines where - despite transcriptional up-regulation of lignin biosynthetic genes (*GmLAC7/12*) - no significant improvement in viral resistance was observed. This paradox suggests that shade-induced resource limitations (particularly reduced photosynthate availability) constrain the metabolic flux required for lignin polymerization, preventing the translation of gene expression into effective physical barriers.

### 3.11 *miR399j* modulates soybean viral defense through light-dependent phosphorus remodeling

Under optimal light conditions, *miR399j* functions as a positive regulator of soybean mosaic virus (SMV) resistance, in contrast to the negative regulatory role of *miR397a* (**Figure 11**). Molecular dissection revealed that *miR399j* directly targets the high-affinity phosphate transporter gene *GmPHT1-4* (GLYMA_10G036800), a key modulator of phosphate (Pi) allocation. This interaction was validated by significant up-regulation of GmPHT1–4 upon *miR399j* inhibition (**Figures 12B, D**).

#### 3.11.1 Inorganic phosphorus content in leaves

Under shaded conditions, SMV infection induced a significant increase in leaf inorganic phosphorus content in both miR399j-overexpressing (OE) lines and empty vector controls (**Supplementary Figure S7A**). However, the magnitude of this increase was significantly smaller in OE lines, suggesting that *miR399j* overexpression helps buffer excessive Pi accumulation in leaves under combined shade and viral stress. Conversely, silencing *miR399j* led to a marked decrease in leaf Pi levels upon infection—contrasting with the Pi increase observed in controls—further supporting *miR399j*’s role as a positive regulator of leaf phosphorus homeostasis (**Supplementary Figure S7B**). Under normal light, SMV infection triggered a significant rise in leaf Pi in OE lines but a decline in controls, with OE plants accumulating substantially more Pi than controls (**Supplementary Figure S7C**). In contrast, both silenced lines and controls exhibited reduced leaf Pi under infection, indicating that *miR399j* activity is required for Pi maintenance in leaves specifically under optimal light (**Supplementary Figure S7D**).

#### 3.11.2 Inorganic phosphorus content in stems

In shade, SMV-infected OE lines showed greater stem Pi accumulation than infected controls, demonstrating that *miR399j* enhances Pi retention in stems even under low light (**Supplementary Figure S7E**). Silenced lines also exhibited increased stem Pi relative to controls under shade—a pattern potentially reflecting altered Pi partitioning rather than enhanced uptake (**Supplementary Figure S7F**). Under normal light, OE lines again displayed superior Pi accumulation in stems post-infection (**Supplementary Figure S7G**), whereas silenced lines suffered a pronounced Pi loss compared to controls, reinforcing *miR399j*’s positive role in stem phosphorus metabolism (**Supplementary Figure S7H**).

#### 3.11.3 Inorganic phosphorus content in roots

Critically, in both shaded and normal light conditions, *miR399j* overexpression consistently promoted root Pi accumulation following SMV infection, with OE lines showing significantly higher Pi levels than controls (**Supplementary Figures S7I, K**). Conversely, *miR399j* silencing resulted in reduced root Pi under both light regimes, with the decline being more severe than in infected controls (**Supplementary Figures S7J, M**). These findings establish *miR399j* as a robust positive regulator of root phosphorus status during viral stress.

**Figure 13:**
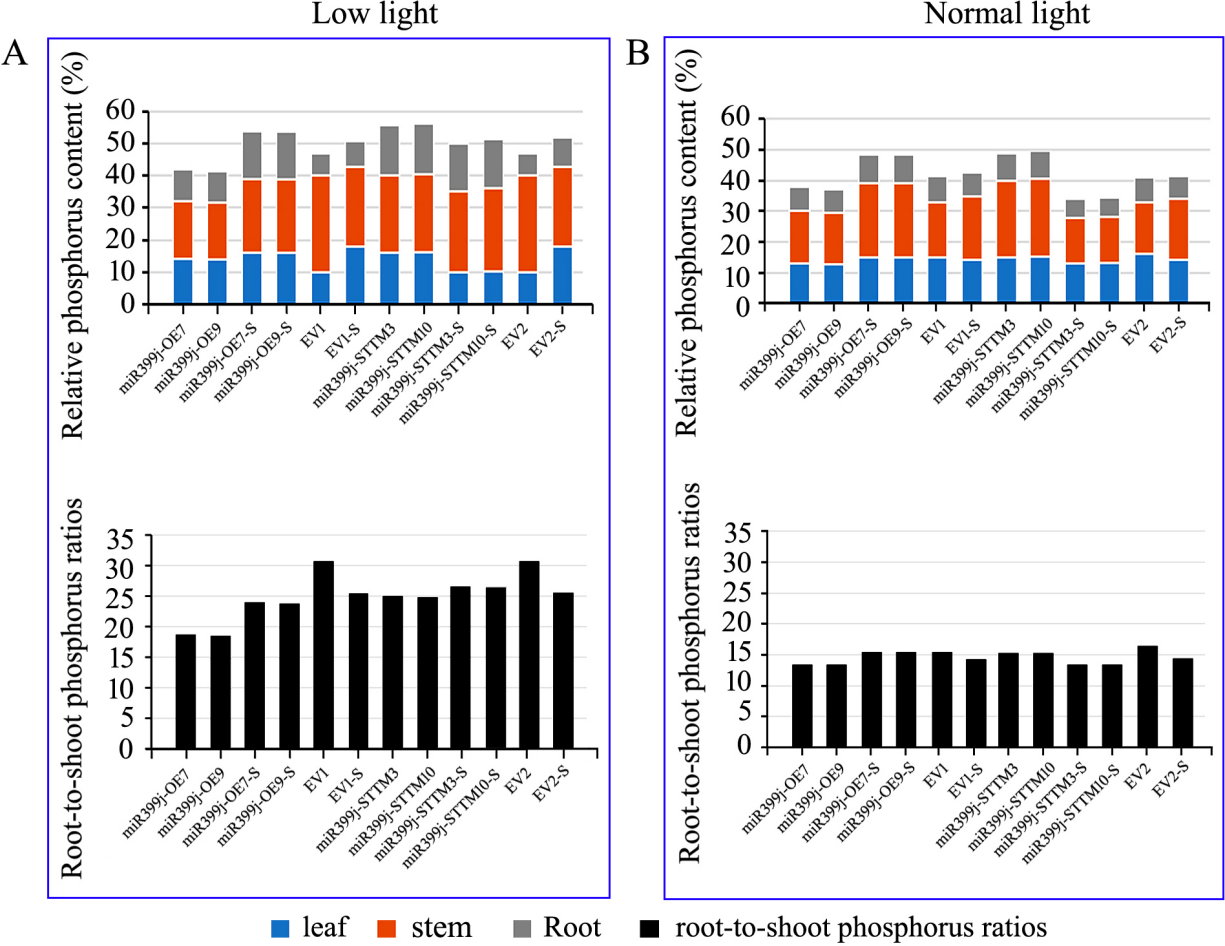
Phosphorus relative content and tissue partitioning in transgenic lines under differential light conditions. **(A)** Phosphorus relative content and tissue partitioning in transgenic lines under low light; **(B)** Phosphorus relative content and tissue partitioning in transgenic lines under normal light. miR399j-OE: the lines overexpressing *miR399j*; miR399j-STTM: the lines with silenced *miR399j*; miR399j-OE-S: the lines overexpressing *miR399j* after virus inoculation; miR399j-STTM-S: the lines with silenced *miR399j* after virus inoculation; EV, Empty vector control plants for the miR399j-OE lines or miR399j-STTM lines; EV1-S and EV2-S, Empty vector control plants after virus inoculation for the miR399j-OE line or miR399j-STTM line; The empty vector control plants (EV1 and EV2) are represented by bars positioned to the right of the mutant bars for visual comparison.

Surprisingly, although *miR399j* overexpression suppressed *GmPHT1-4*, it was associated with elevated inorganic phosphorus (Pi) content in roots, stems, and leaves under normal light conditions (**Supplementary Figures S7C, G, K**; **Figure 13B**). This suggests that *miR399j* may activate compensatory mechanisms—such as up-regulation of alternative PHT transporters or enhanced intracellular Pi remobilization—that collectively sustain tissue Pi accumulation despite reduced *GmPHT1–4* expression. Conversely, *miR399j* silencing increased *GmPHT1–4* transcript abundance but consistently lowered Pi levels across all tissues under both normal and shaded conditions (**Supplementary Figures S7D, H, M**; **Figure 13**), highlighting complex post-transcriptional regulation and functional redundancy within the PHT1 family.

A critical light-dependent shift occurs in phosphorus allocation and defense strategy, wherein *miR399j* reconfigures Pi distribution to prioritize shoot-based immunity under normal light and root-centered antiviral functions under shade. Under shade stress, *miR399j* activity drives a tissue-specific reconfiguration of phosphorus distribution. Quantification revealed higher root-to-shoot Pi ratios in miR399j-OE lines compared to controls (**Supplementary Figures S7A, E, I, B, F, J**; **Figure 13A**), indicative of enhanced root phosphorus retention. Notably, this redistribution coincided with significantly reduced SMV accumulation in roots of OE plants under shade (**Figure 11**), suggesting that root-localized Pi enrichment contributes to viral containment. In contrast, miR399j-silenced lines exhibited diminished root Pi and disrupted phosphorus homeostasis, correlating with compromised antiviral outcomes. Importantly, our data indicate that light-modulated phosphorus allocation modulates—but does not universally suppress—antiviral capacity. Rather than reflecting a maladaptive trade-off, the observed Pi reallocation appears to be part of an integrated physiological strategy that prioritizes resource allocation to critical sinks (e.g., roots) under stress. Supporting this view, we previously reported that shade-induced attenuation of defense signaling—including dampened salicylic acid responses and moderated ROS bursts—can paradoxically restrict SMV replication by limiting the metabolic substrates required for viral propagation (Shang et al., 2023). Thus, miR399j-mediated phosphorus remodeling likely functions as a context-dependent adaptive mechanism that optimizes resource use under combined biotic and abiotic stress.

### 3.12 Phosphorus availability determines lignin-dependent antiviral defense under shade

Under shade conditions, *miR397a*-silenced soybean plants exhibited up-regulated expression of laccase genes *GmLAC7* and *GmLAC12*, yet failed to accumulate lignin in leaves and showed enhanced susceptibility to SMV. To test whether this defense defect was due to insufficient phosphorus (Pi) availability, we applied exogenous Pi via foliar spray. As shown in **Supplementary Figure S8**, Pi supplementation significantly restored leaf lignin content in shaded miR397a-silenced plants, concomitant with a marked reduction in SMV RNA accumulation compared to Pi-untreated controls. These results indicate that Pi availability is a critical determinant of lignin biosynthesis and antiviral defense efficacy under low-light stress.

Our findings delineate a light-gated regulatory axis through which *miR399j* fine-tunes phosphate (Pi) homeostasis to modulate antiviral defense. *miR399j* directly suppresses *GmPHT1-4*, a key high-affinity Pi transporter, yet this repression triggers systemic compensation via alternative transport pathways that sustain Pi acquisition under normal light, thereby enhancing SMV resistance. Under shade stress, however, the same regulatory circuit shifts toward environment-responsive phosphorus allocation: Pi is preferentially retained in roots—a pattern strongly associated with reduced SMV accumulation in root tissues of *miR399j*-overexpressing lines—while Pi availability in shoots is markedly reduced. This reallocation critically constrains defense execution in aerial tissues. Specifically, in *miR397a*-silenced plants under shade, although *GmLAC7* and *GmLAC12* are transcriptionally up-regulated, limited leaf Pi availability restricts carbon flux into the phenylpropanoid pathway, preventing effective lignin polymerization. Consequently, structural barriers fail to form, facilitating viral spread. Critically, exogenous Pi supplementation rescues lignin deposition and suppresses SMV accumulation under shade (**Supplementary Figure S8**), confirming that Pi—not laccase expression alone—is the limiting factor for defense output. Thus, *miR399j* functions as a dual-output regulator—promoting Pi-dependent immunity under optimal light while reconfiguring Pi distribution to prioritize root-based antiviral capacity under shade—highlighting how light conditions critically determine the functional interplay between miRNA-mediated nutrient regulation and plant immunity.

## 4 Discussion

In soybean intercropping systems, a reduced red-to-far-red light ratio (R:FR) triggers shade avoidance responses, significantly decreasing biomass and root-to-shoot ratio while promoting stem elongation (Yang et al., 2014). Under low R:FR, carbon is preferentially allocated to shoots at the expense of root development, reducing total root length, surface area, and volume (Lyu et al., 2023). Concurrently, Soybean mosaic virus (SMV) infection induces leaf mottling and crinkling (Jiang T. et al., 2023), further inhibiting growth and compromising root architecture (Chen et al., 2017). In our study, shaded soybean plants exhibited reduced root length and a lower root-to-shoot ratio by 10 days post-infection (dpi), consistent with synergistic effects of shade and viral stress on resource allocation. Notably, early SMV accumulation (3 dpi) was lower under shade, likely reflecting slowed viral replication due to reduced photosynthetic activity or growth prioritization—a transient effect that reverses during sustained infection as defense capacity wanes.

Phosphate (Pi), the primary bioavailable form of phosphorus, sits at the nexus ofgrowth–defense trade-offs. While shading can enhance total P uptake in legumes such as red clover (Xu et al., 2021), it often diverts P away from yield-related sinks (Patel et al., 2021). SMV, in turn, hijacks host nutrients to support its replication (Jiang and Zhou, 2023), potentially disrupting systemic P partitioning (Díaz-Cruz and Cassone, 2018). In our dual-stress system, P distribution shifted dynamically: during early infection (Days 1–5), P accumulated preferentially in leaves > roots > stems; by Days 6–11, leaf P pools stabilized despite ongoing viral challenge. Both shade and SMV independently stimulated early Pi uptake, with shade driving greater P accumulation in leaves and infected roots than under normal light—consistent with a short-term investment in morphological acclimation. However, by late infection, this allocation pattern constrained defense execution, revealing a temporal hierarchy where plasticity for light capture precedes—but ultimately compromises—antiviral immunity under prolonged abiotic stress (Cipollini et al., 2018).

Hormonal signaling further modulates this trade-off. Low R:FR suppresses jasmonic acid (JA) signaling while elevating auxin and gibberellins to promote shade avoidance (de Wit et al., 2013; Roeber et al., 2021). In line with this, we observed attenuated induction of *GmNPR1-1*—a central regulator of salicylic acid (SA) signaling—under shade at 3 dpi, likely due to reduced NPR1 phosphorylation (Shang et al., 2023). Although GmPR1-6, a positive regulator of SMV resistance, was strongly upregulated in leaves and roots early in infection, its expression declined in roots by 10 dpi, suggesting that SA-mediated defenses are primarily active during initial pathogen recognition. This aligns with KEGG analyses: under normal light, SMV infection initially upregulates photosynthesis and carbon metabolism (Day 3), while suppressing phenylpropanoid biosynthesis and plant–pathogen interaction pathways. By Day 10, however, phenylpropanoid biosynthesis is robustly induced, supporting the production of lignin and flavonoids (Yuan et al., 2019)—processes critically dependent on Pi-derived energy and precursors.

Under shade, root responses diverge markedly. At 3 dpi, KEGG enrichment reveals induction ofglutathione metabolism, α-linolenic acid metabolism, and zeatin biosynthesis—pathways linked to antioxidant defense and stress tolerance. Yet GO analysis simultaneously shows down-regulation of heme binding and antioxidant activity (MF), hinting at an initial but unsustainable defense effort. By 10 dpi, antioxidant capacity declines further, while calcium ion binding increases (GO-MF), coinciding with activation of plant–pathogen interactions and MAPK signaling (KEGG). This calcium surge may activate calcium-dependent protein kinases in MAPK cascades (Xu et al., 2022), potentially compensating for Pi starvation–induced calcium dysregulation (Matthus et al., 2019). Nevertheless, compromised photosynthesis (GO/KEGG) and suppressed carbon fixation limit substrate availability for defense metabolites. Increased root inorganic P under shade may support glutathione synthesis and acid phosphatase secretion (Zhang et al., 2022), aiding P scavenging, while JA-mediated P utilization (Sun et al., 2023) and MAPK-coordinated stress responses (Khan et al., 2023) contribute to shade adaptation—orchestrated in part by soybean shade-tolerance genes (Jiang A. et al., 2023). Nonetheless, these adjustments prioritize survival over immunity, consistent with impaired SA/JA defenses in Arabidopsis under low R:FR (Fernández-Milmanda et al., 2020).

A key insight from our work concerns the post-transcriptional regulation of structural immunity. Pathogen challenge typically down-regulates *miR397*, enhancing laccase activity and promoting lignin deposition to reinforce cell walls—a conserved mechanism observed in cotton (GhLAC4; Wei et al., 2021), apple (MhLAC7/LAC17; Yu et al., 2020), pear (PcLACs; Yang et al., 2023), and other hosts during viral infection (Li et al., 2017; Xia et al., 2018). We confirm that soybean *miR397a* directly targets GmLAC7 and GmLAC12, positioning it as a negative regulator of lignin-based antiviral defense. However, silencing *miR397a* fails to enhance resistance under shade despite elevated GmLAC7/12 expression, revealing a critical metabolic bottleneck: lignin biosynthesis requires not only enzyme transcription but also sufficient leaf Pi pools to fuel the phenylpropanoid pathway and regenerate redox cofactors. Shade-induced systemic P reallocation to roots depletes Pi in leaves by 25–40%, uncoupling gene expression from functional immunity.

Critically, exogenous Pi application restores lignin deposition and suppresses SMV accumulation under shade (**Supplementary Figure S8**), establishing a causal link between leaf Pi availability and structural defense output. This demonstrates that Pi’s role in immunity is context-dependent: while it may modulate hormone signaling, it is indispensable for energy-intensive physical barriers like lignin.

Beyond its canonical role in Pi homeostasis via PHO2 suppression (Zhang et al., 2019; Zhao et al., 2013), we show that *miR399j* functions as a light-gated regulator of growth–defense balance by targeting the high-affinity Pi transporter GmPHT1-4 (**Figure 9B**). Under normal light, *miR399j* overexpression reduces root:shoot Pi ratios and enriches leaf Pi pools, providing substrates (ATP, nucleotides, polyphosphates) for defense metabolism and lowering SMV titers. In contrast, shade increases root:shoot Pi ratios by 58–100% across genotypes—a shift driven by light-responsive elements in the GmPHT1–4 promoter (Wei et al., 2022)—thereby limiting carbon flux into lignin biosynthesis and impairing ROS-mediated defenses. This mechanism unifies prior observations of phosphite-activated immunity (Vinas et al., 2020) and SA/JA induction (Val-Torregrosa et al., 2022) into a spatial model: under optimal light, *miR399j* directs Pi toward aerial tissues to potentiate defense; under shade, resource conservation in roots takes precedence, sacrificing inducible immunity for metabolic maintenance.

In summary, our findings redefine the plant’s response to combined shade and viral stress not as a simple hormonal trade-off, but as a metabolically gated decision governed by the subcellular and tissue-level allocation of a limiting nutrient—phosphorus. Optimizing crop resilience in heterogeneous light environments will thus require strategies that decouple Pi conservation from defense suppression, such as engineering light-insensitive PHT1 variants or modulating *miR399j* activity in a tissue-specific manner.

## 5 Conclusion

This study deciphers how soybean prioritizes growth-defense investments under variable light and viral stress, revealing that shade-induced low R: FR ratios trigger systemic carbon reallocation and phosphorus bottlenecks, elevating root: shoot P ratios and reducing leaf Pi availability, which uncouple transcriptional defense programming from functional outputs, exemplified by failed lignin deposition in *GmLAC7/12*-up-regulated *miR397a*-silenced lines. Crucially, we identify antagonistic miRNA hubs: *miR397a* promotes SMV susceptibility via laccase repression, while *miR399j* enhances resistance by remapping phosphorus allocation through *GmPHT1–4* targeting, reducing root: shoot ratios and directing more P to leaves to fuel defense metabolism—yet both pathways are overridden under shade, forcing a light-gated resource hierarchy that sacrifices immunity for metabolic maintenance. These findings establish a paradigm where environmental control of miRNA-directed phosphorus spatial distribution and carbon flux dictates stress resilience, providing a mechanistic blueprint for developing soybean varieties with optimized partitioning traits in dynamic cropping systems.

## Data Availability

Publicly available datasets were analyzed in this study. This data can be found here: The datasets presented in this study can be found in online repositories (https://ngdc.cncb.ac.cn/bioproject/browse/PRJCA040278). The names of the repositories and accession number can be found below: CNCB accession number PRJCA040278.
