# Supplementary material for: Balancing growth and defense: miRNA-mediated regulation of phosphorus allocation and antiviral immunity in soybean under normal light and shade
Source: Front Plant Sci. 2026 Mar 30;16:1707038. doi: 10.3389/fpls.2025.1707038 (PMC13071050; doi:10.3389/fpls.2025.1707038)
Supplement: Supplementary file 1 [file Table1.docx]

*Supplementary_Material*

**Balancing Growth and Defense: miRNA-Mediated Regulation of Phosphorus Allocation and Antiviral Immunity in Soybean Under Normal Light and Shade**

Jing Shang^†*^, Xinmiao Yang^†^, Siyu Li, Jidan Hu, Lingfang Du and Wenyu Yang

Sichuan Engineering Research Center for Crop Strip Intercropping System and College of Agronomy, Sichuan Agricultural University, Chengdu 611130, China

*** Correspondence:** Jing Shang; shangjing_edu@163.com

† These authors contributed equally to this work.

**Table S1 Light conditions of different experimental groups**

| Treatment | Light intensity  (μmol·m-2·s -1) | Light quality  (μmol·m-2·s -1) |
| --- | --- | --- |
| NC | 565.14 | 4.27 |
| NS | 565.14 | 4.27 |
| LC | 50.85 | 0.48 |
| LS | 50.85 | 0.48 |

**Table S2 Primers for RT-qPCR**

| Gene ID | Forward primer | Reverse primer |
| --- | --- | --- |
| SMV-670 | AAAGAGAGGGTAACACACAA | GATCGCTCATCAAACAATAA |
| Actin | CCATGTTCCCTGGTATTGCTG | GTATTTTCTCTCCGGTGGTGC |
| SMV-CP | GCTTGGACCACTTGCT | ACACCCATCTGCTCATC |
| GmNPR1-1 | CTGGTCTTTCTGCGTCAA | CGTAAGGGCTTCCATTCT |
| GmPR1-6 | TGGTGACCTAAGTGGCACAG | GCAGACACTCTCCACCAACA |
| GmMYC2 | GCTTTAGGCTCGGTTCTC | GTGCTGTACGAGGTTCCA |
| GmPDF1.2 | GCAAACTGAGGCAAAGAC | GCACCAACAGCGAAAATC |
| GmERF1A | AAATCAAGCACCACTCAA | CACCTCAGCCTGCAACTC |
| GmERF1B | ATGGAAAGCGACAGGAAT | TAGCGTCAACCGAAGAGG |
| GLYMA_01G010700 | GCTGCGTATGCTCAAGAC | CACCACCAGAGTGGACAA |
| GLYMA_01G091800 | GAGGGGCAACTCATTCCAGA | AGCTTGGTGCTTGTTTTGTGTA |
| GLYMA_02G005800 | GACAAGATAGGATGTGAATGC | CCATCCCAAGAATGTATTGC |
| GLYMA_03G189200 | AGGGTCCATGTTTAGAGCCG | TCTTTTGCTTTCTGAGCCGC |
| GLYMA_07G002900 | GACCACTCTAGCATCACCTG | TTCTGGTGGAATAGGTGGAAG |
| GLYMA_08G021900 | TTGATGGGATCTACAACAGGTC | ATCAATTTCCACCGGATCAAG |
| GLYMA_09G022600 | GTTTGATCCTTCGGTTGGTG | ATTTGGCCCCATAAACCTCT |
| GLYMA_10G036800 | TGTCACTACGTCTACTCTTGATGT | CGTCTTGCTGAGTCATGTATGC |
| GLYMA_20G163400 | CTTCGTTCTTCATTTGCGTA | TGATTAAGGTACGCTCGATAG |
| GLYMA_20G204100 | CTCCCAGCTCTGCTCACATA | CCAACAGGAACCAAGTAGTGGC |
| U6 | GACCATTTCTCGATTTGTGCGTGTC | Universal reverse Q primer  Universal reverse Q primer |
| gma-miR1512b | GGCCGCCGTAACTGGAAATTCTTAAAGCAT |  |
| gma-miR159d | GGCAGCTGCTTAGCTATGGATCCCA |  |
| gma-miR167a | TGAAGCTGCCAGCATGATCTAA |  |
| gma-miR167e | CCTGAAGCTGCCAGCATGATCTTA |  |
| gma-miR3522 | GAGACCAAATGAGCAGCTGA |  |
| gma-miR397a | TGAAGCTGCCAGCATGATCTAA |  |
| gma-miR398c | GTGTGTTCTCAGGTCGCCCCTGAA |  |
| gma-miR408d | GCTGCACTGCCTCTTCCCTGGAA |  |
| gma-miR4365 | AAGAACTTCTTCCGCGAGATCGCA |  |
| gma-miR5380c | ATGAATGGTGAAGATGAAGAG |  |
| pCAMBIA1300-35S-EGFP | CGTGGAAAAAGAAGACGTTCCAACCAC | CGTCGCCGTCCAGCTCGACCAG |
| pGreenII 0800-miRNA | TAACAACCGCGAAAAAGTTGCG | AGATCTCAAACAAACACATACAGCG |
| Pre-miR397a | acgggggacgagctcggtaccCGATAGATAATAGTAACGACTAGTCAATTAGTG | cgcgtacgagatctggtcgacTGGTTCCTGAAATAAAACTGCTCA |
| Pre-miR399j | acgggggacgagctcggtaccATGCTTATCAGTGGCATATCCAAG | cgcgtacgagatctggtcgacCCAATTGTCTCAGAAAATCTCTGATT |
| Pre-miR408d | acgggggacgagctcggtaccTATATATAGAGAGGGATTATATCAAATCTATGC | cgcgtacgagatctggtcgacGCCGGGCAACTGTGGATG |
| GmPHT1-4 | ttctagagcggccgcggatccGCCGTGCCTTAATTCGGC | actggtgatttcagcgaattcCACAGTAACACCATAATAAACCGAGC |
| GmLAC7 | ttctagagcggccgcggatccTTGTGAGTTATCGAGTTAAACCTTCAA | actggtgatttcagcgaattcTGTTGATTGAAACGCCTGGG |
| GmLAC12 | ttctagagcggccgcggatccCGAAACACCCATTCTTCTTGGG | actggtgatttcagcgaattcATGAGGTGACTTGTATTCGAGTATGG |
| GmNUD2 | ttctagagcggccgcggatccATGATGAGTTCTAGAGCTTGTAAGCC | actggtgatttcagcgaattcTGGTGGAATCTGAATCCAGCC |
| GmPHT1-4-MUT | TtctcattaataagaaGACCACGAAGTTTAATTCATTCTTCT | CttcttattaatgagaAATGATCTAATTCAGCAGGAAGATTATAG |
| GmLAC7-MUT | CTGCAgagttacCACAACACTTTTTTAAGATAGCCAACC | TTGTGgtaactcTGCAGCGTTGATAATGCGCA |
| GmLAC12-MUT | CTGCAgagttagtACCTCTCTTCTTCACCGTCGC | GAGGTactaactcTGCAGCATTGATAACACGTAGAAG |
| GmNUD2-MUT | AAGAGcttcacgCAAGTAAAGCCATTGATACACTCAGA | ACTTGcgtgaagCTCTTCCTTGGCCAGAGAAGC |
| RT-U6 | GTGCAGGGTCCGAGGTTTTGGACCATTTCTCGAT | |
| RT-miR399j | GTCGTATCCAGTGCAGGGTCCGAGGTATTCGCACTGGATACGACCAGGGC | |
| RT-miR397a | GTCGTATCCAGTGCAGGGTCCGAGGTATTCGCACTGGATACGACCATCAA | |
| RT-miR408d | GTCGTATCCAGTGCAGGGTCCGAGGTATTCGCACTGGATACGACGCCAGG | |
| q-U6 | GGAACGATACAGAGAAGATTAGCA | GTGCAGGGTCCGAGGT |
| q-miR397a | GGCTTACGTCATTGAGTGCAGC | GTGCAGGGTCCGAGGT |
| q-miR399j | CGCGTGCCAAAGGAGATTT | GTGCAGGGTCCGAGGT |
| q-miR408d | CCACCATGTGCACTGCCTCTT | GTGCAGGGTCCGAGGT |
| linker-STTM | GTTGTTGTTGTTATGGTCTAATTTAAATATGGTCTAAAGAAGAAGGAAT | |
| STTM-miR399j | GGAGAGGACAGGGTACCCcagggcaaatcctatcctttggcaGTTGTTGTTGTTATGG | CTCTAGAGGATCCCCtgccaaaggactagatttgccctgATT CTTCTTCTTTAGACCA |
| STTM-miR397a | GGAGAGGACAGGGTACCCCATCAACGCTGCTACACTCAATGAGTTGTTGTTGTTATGG | CTCTAGAGGATCCCCTCATTGAGTGCTACAGCGTTGATGATTCTTCTTCTTTAGACCA |
| 5'RACE adaptor | GCUGAUGGCGAUGAAUGAACACUGCGUUUGCUGGCUUUGAUGAAA | |
| RACE Outer Primer | GCTGATGGCGATGAATGAACACTG | |
| 5'RACE Inner Primer | CGCGGATCCGAACACTGCGTTTGCTGGCTTTGATG | |
| GmLAC7 gene-specific outer primer | TGGCATTCTCATAAATGACCAC | |
| GmLAC7 gene-specific inner primer | TGGAGTGAAGGCCATGTAAT | |
| GmLAC12 gene-specific outer primer | ATGGTGAGAATGATTACT | |
| GmLAC12 gene-specific inner primer | AGGTGACTTGTATTCGAGTA | |
| GmPHT1-4 gene-specific outer primer | ATGGTGGCCGAAAGAGGG | |
| GmPHT1-4 gene-specific inner primer | TCTCCACCAATGCCAAAACCA | |
| GmNUDT2 gene-specific outer primer | TCCAATTCACAAGCATCAAGTAAT | |
| GmNUDT2 gene-specific inner primer | ATGGTGGAATCTGAATCCAGCCTTAA | |


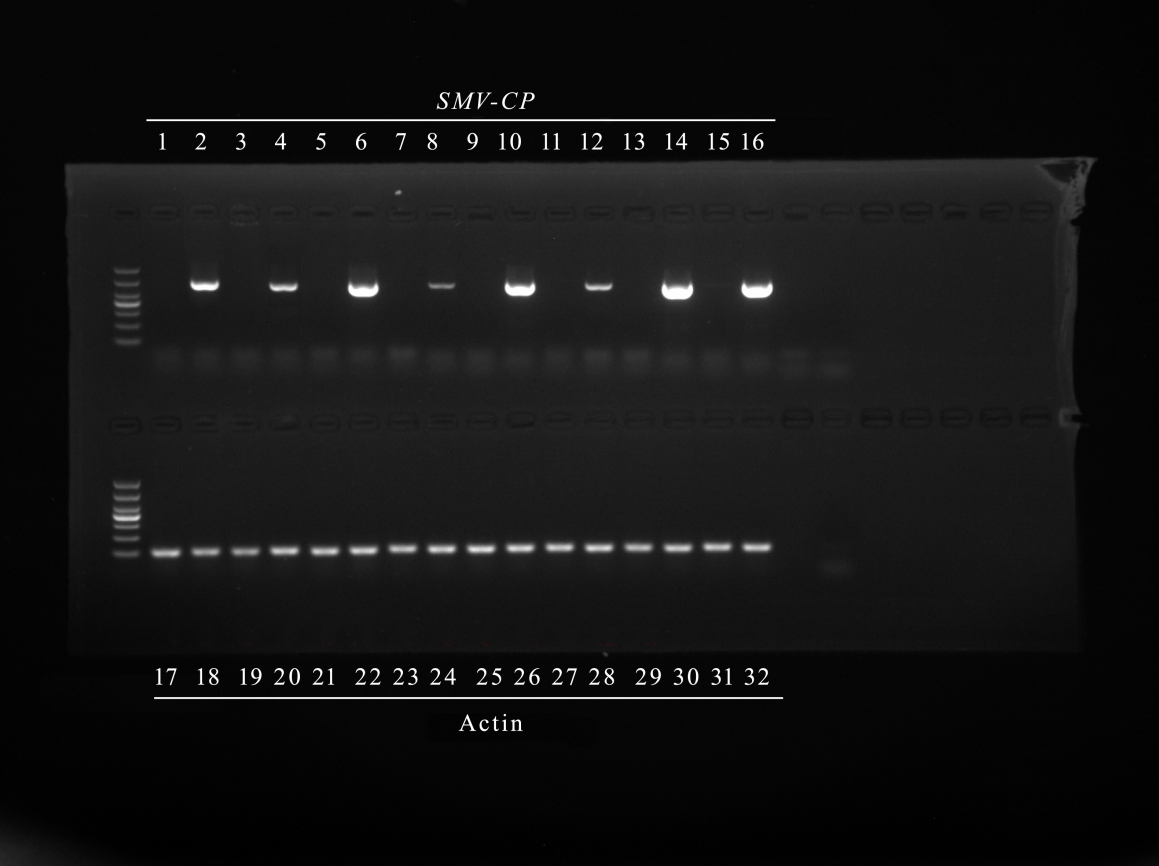


**Figure S1 The original image of the gel corresponding to Figure 1C in the main text.** Note: the figure shows viral replication in soybean plants under shaded and normal light conditions in leaves and roots (the upper part of the figure), the lower part of the figure shows the electrophoresis results of ACTIN after quantification. Lanes 1-4: Viral content detection in soybean leaves from NC, NS, LC, and LS treatment groups at 3 days post inoculation (dpi); Lanes 5-8: Viral content detection in soybean roots from NC, NS, LC, and LS treatment groups at 3 dpi; Lanes 9-12: Viral content detection in soybean leaves from NC, NS, LC, and LS treatment groups at 10 dpi; Lanes 13-16: Viral content detection in soybean roots from NC, NS, LC, and LS treatment groups at 10 dpi.

**
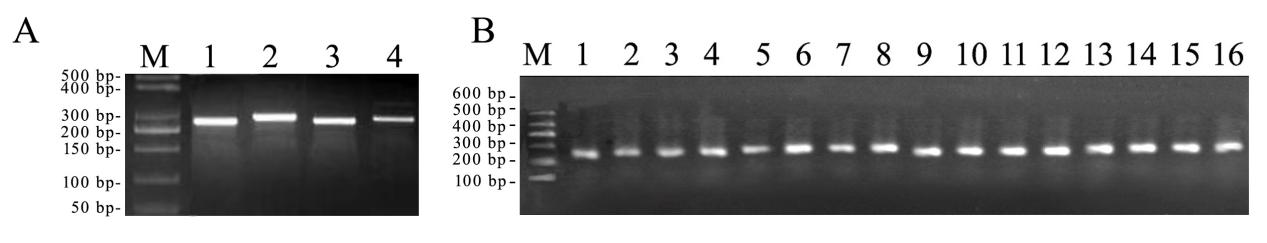
**

**Figure S2 Validation of miRNA-Mediated Cleavage Sites by RLM-RACE Assay.** Note: A, Electrophoresis detection of the second-round PCR products; bands 1-4 are *GmLAC7*, *GmLAC12*, *GmPHT1-4*, and *GmNUDT2*, respectively; B: Colony PCR detection; bands 1-4 are *GmLAC7*; bands 5-8 are *GmLAC12*; bands 9-12 are *GmPHT1-4*; and bands 13-16 are *GmNUDT2* (Original gel images are showed in Figure S3).

**
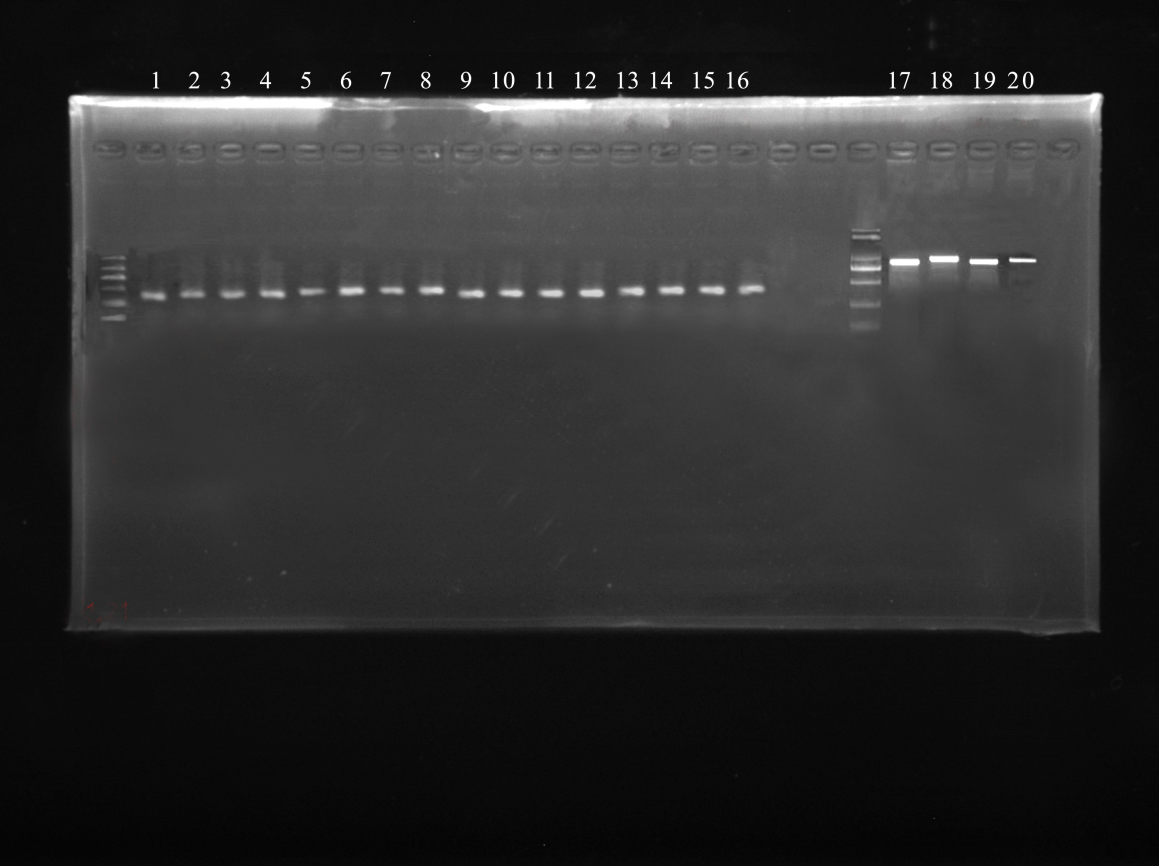
**

**Figure S3 The original image of the gel corresponding to Figure S2.** Note: bands 17-20 are electrophoresis detection of the second-round PCR products; bands 17-20 are *GmLAC7*, *GmLAC12*, *GmPHT1-4*, and *GmNUDT2*, respectively; bands 1-16 are colony PCR detection; bands 1-4 are *GmLAC7*; bands 5-8 are *GmLAC12*; bands 9-12 are *GmPHT1-4*; and bands 13-16 are *GmNUDT2*.

**
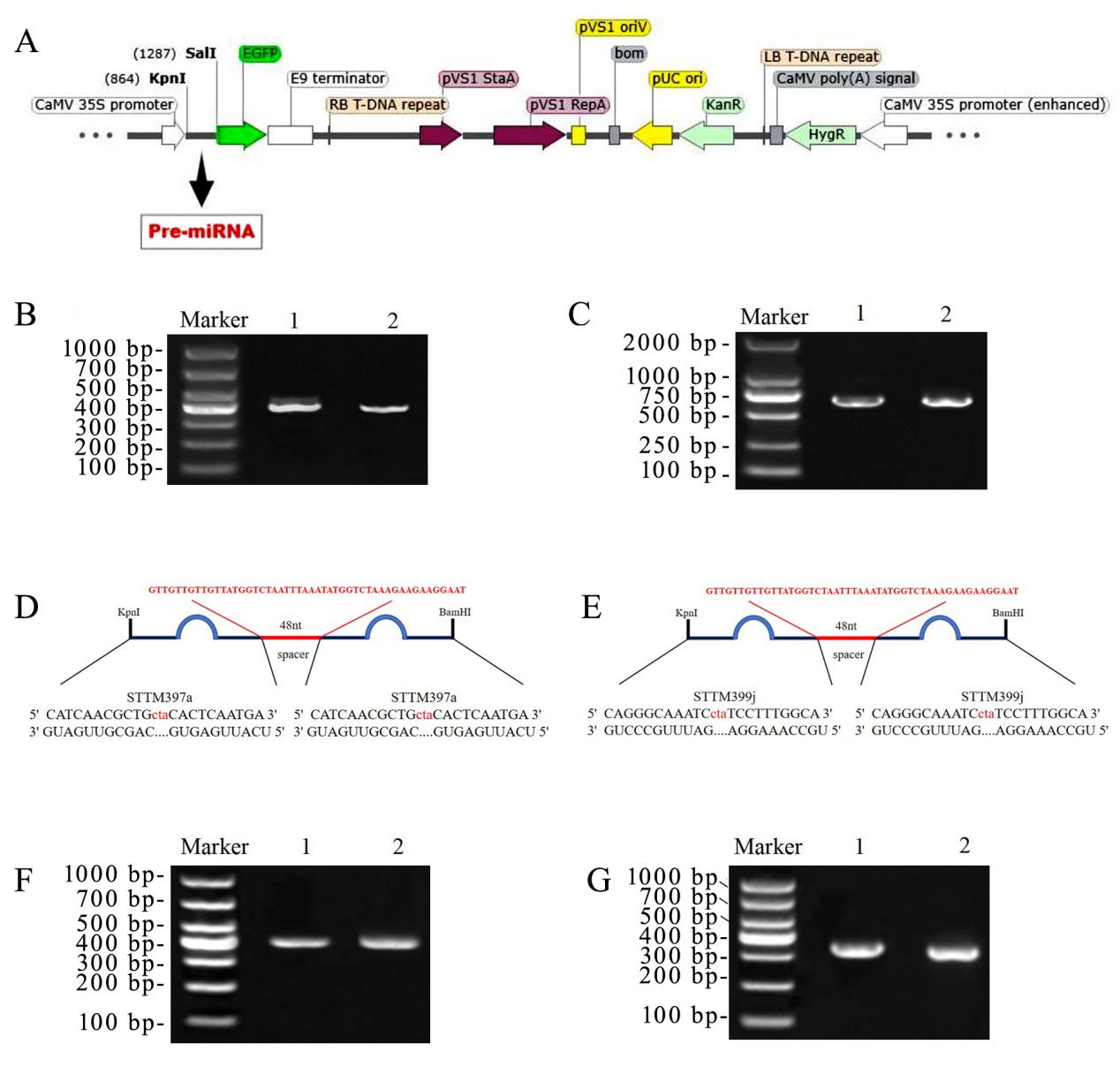
Figure S4.** **Construction of miRNA overexpression and silencing lines.** Note: A: Schematic diagram of overexpression lines construction; B: Amplification of approximately 400 bp miRNA precursor sequences, band order is 1: *miR397a*, 2: *miR399j*; C: E. coli colony PCR electrophoresis detection, band order is 1: *miR397a*, 2: *miR399j*; D: Schematic diagram of the miR397a-STTM vector; E: Schematic diagram of the miR399j-STTM vector; F: Amplification of approximately 400 bp pEasy-STTM-miRNA, band order is 1: *miR397a*, 2: *miR399j*; G: E. coli colony PCR electrophoresis detection, band order is 1: *miR397a*, 2: *miR399j* (Original gel images are showed in Figure S5).


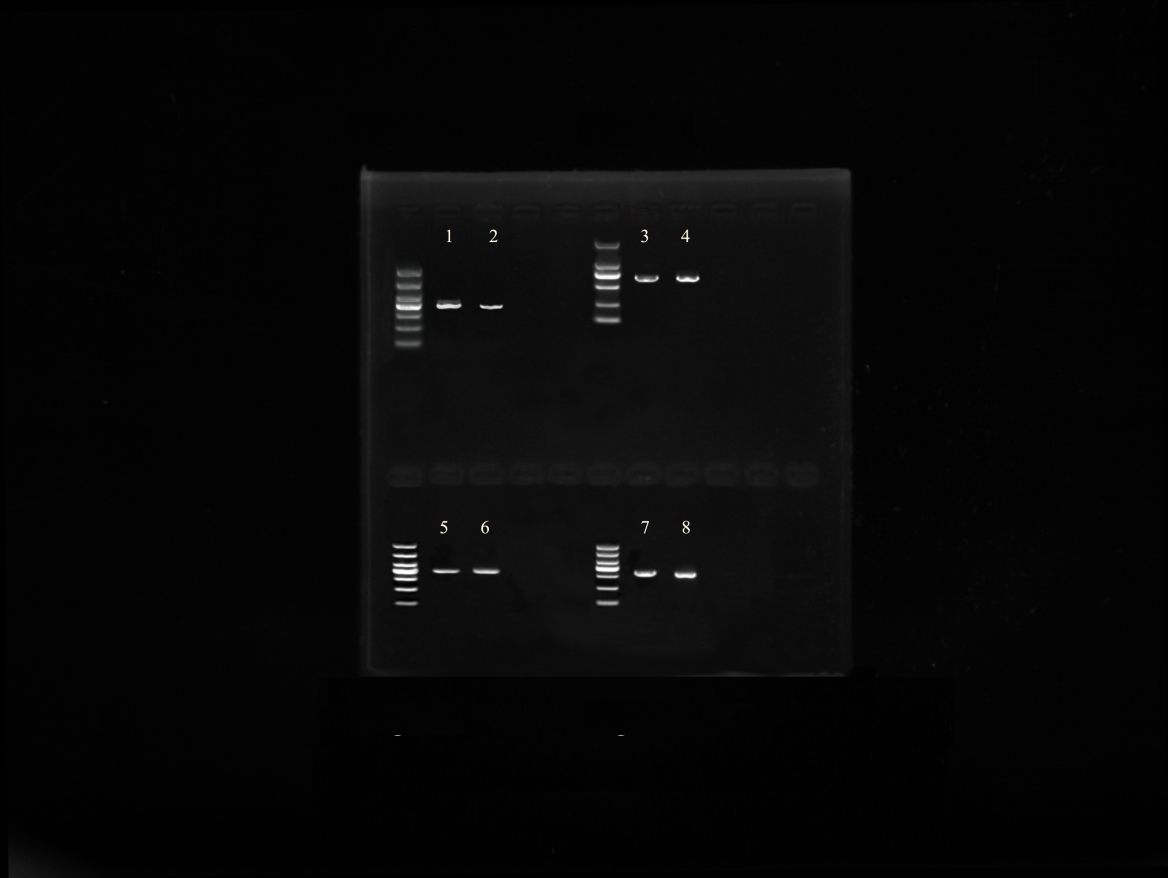


**Figure S5 The original image of the gel corresponding to Figure S5.** Note: 1-2:Amplification of approximately 400 bp miRNA precursor sequences, band order is 1: *miR397a*, 2: *miR399j*; 3-4: E. coli colony PCR electrophoresis detection, band order is 3: *miR397a*, 4: *miR399j*; 5-6: Amplification of approximately 400 bp pEasy-STTM-miRNA, band order is 5: *miR397a*, 6: *miR399j*; 7-8: E. coli colony PCR electrophoresis detection, band order is 5: *miR397a*, 6: *miR399j*.


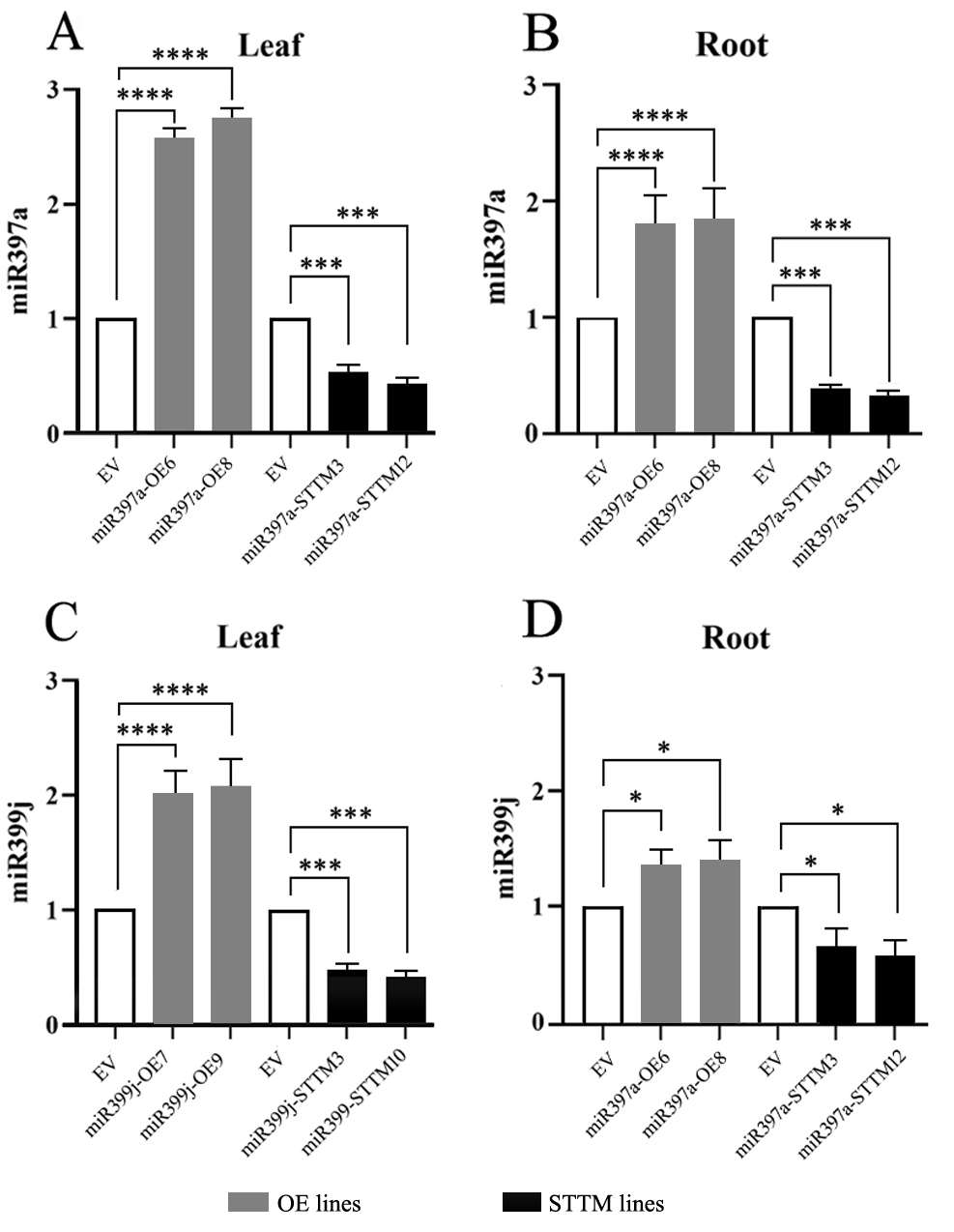


**Figure S6. Detection of silencing and overexpression efficiency of *miR397a* and *miR399j*.** A: Detection of *miR397a* expression in leaves of transient overexpression and silencing *miR397a* lines; B: Detection of *miR397a* expression in root tissues of transient overexpression and silencing *miR397a* lines; C: Detection of *miR399j* expression in leaves of transient overexpression and silencing *miR399j* lines; D: Detection of *miR399j* expression in root tissues of transient overexpression and silencing *miR399j* lines. The empty vector (EV) control plants are represented by bars positioned to the right of the mutant bars for visual comparison (set as 1). Note: data are presented as mean±standard deviation. Statistical significance: *p<0.05, **p<0.01, ***p<0.001, ****p<0.0001.

**
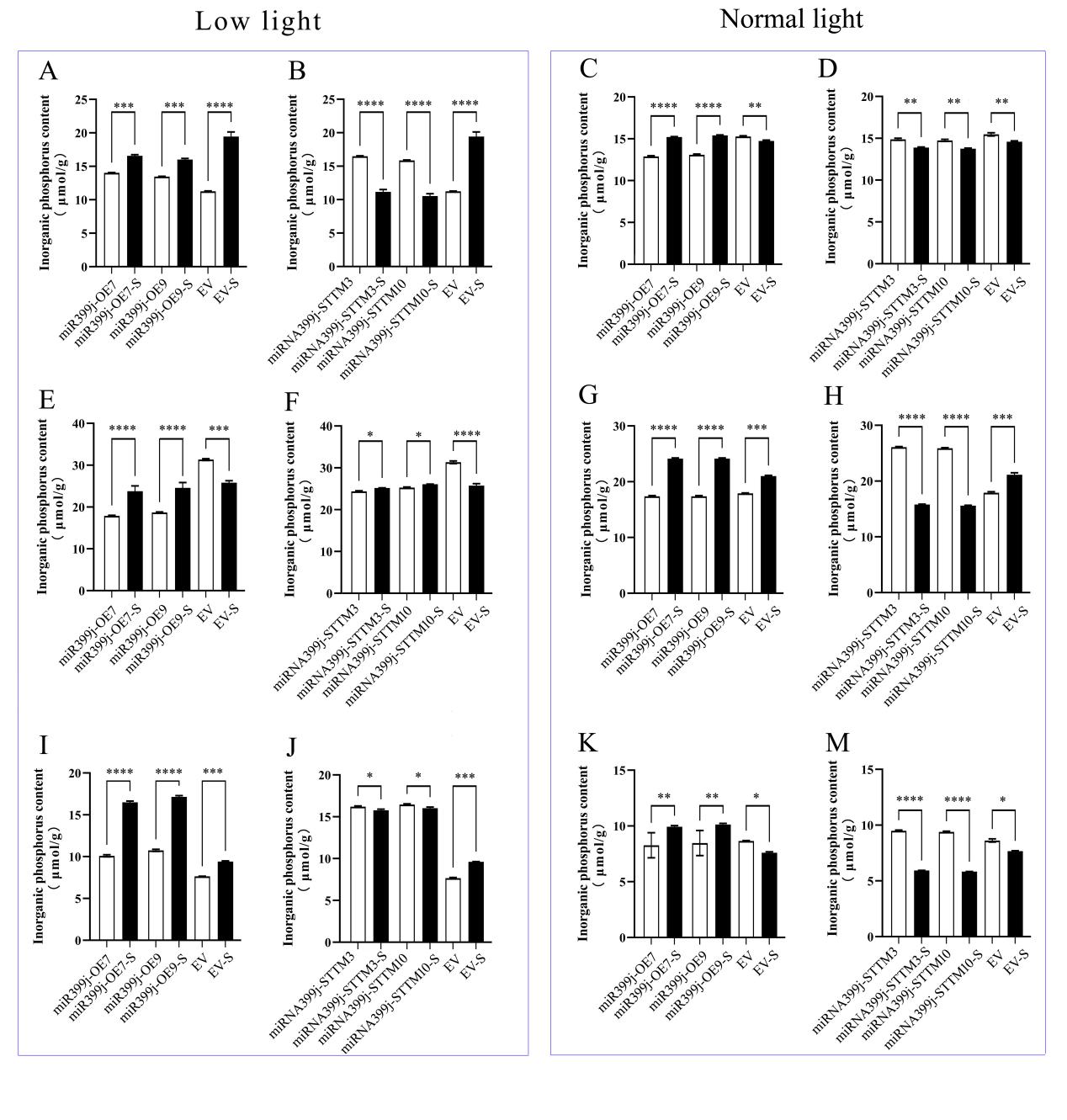
Figure S7.** Changes in inorganic phosphorus content in the *miR399j* mutant. Note: The left frame of the figure shows the detection results of inorganic phosphorus content in the leaves (A and B), stems (E and F), and roots (I and J) of the *miR399j* mutant under shaded conditions; the right frame of the figure shows the detection results of inorganic phosphorus content in the leaves (C and D), stems (G and H), and roots (K and M) of the *miR399j* mutant under normal light conditions; miR399j-OE: the lines overexpressing *miR399j*; miR399j-STTM: the lines with silenced *miR399j*; miR399j-OE-S: the lines overexpressing *miR399j* after virus inoculation; miR399j-STTM-S: the lines with silenced *miR399j* after virus inoculation; EV: Empty vector control plants for the miR399j-OE line or miR399j-STTM line; EV-S: Empty vector control plants after virus inoculation for the miR399j-OE line or miR399j-STTM line; The empty vector (EV) control plants are represented by bars positioned to the right of the mutant bars for visual comparison (set as 1). The data are presented as the means ± standard deviations; * indicates p<0.05, ** indicates p<0.01, *** indicates p<0.001, and **** indicates p<0.0001.

## *
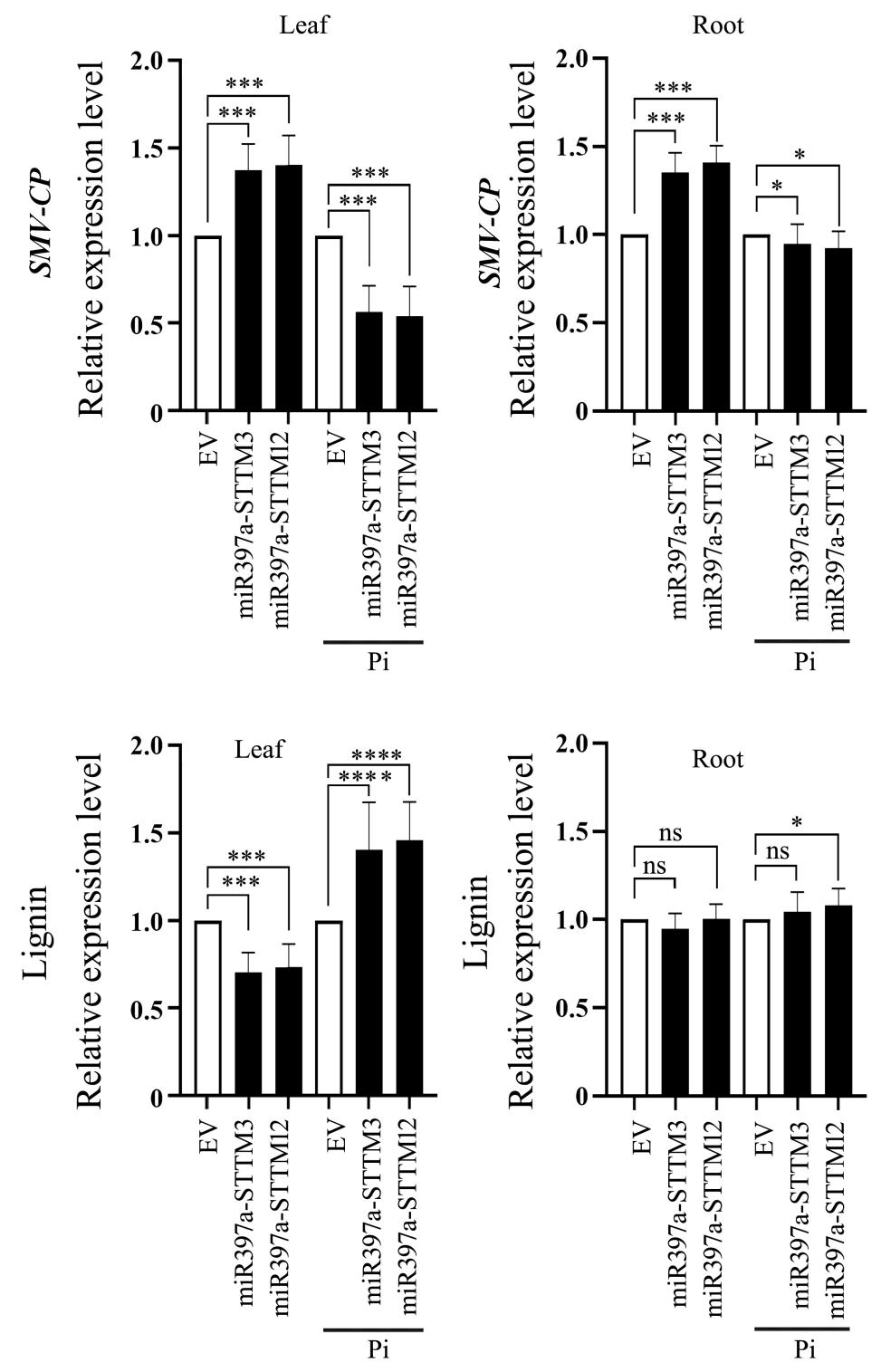
*

**Figure S8.** Phosphorus supplementation restores lignin deposition and suppresses SMV accumulation under shade. Lignin content in leaves of wild-type and miR397a-STTM soybean plants grown under normal light or shade, with or without exogenous inorganic phosphate (Pi) application. SMV RNA accumulation, quantified by RT-qPCR. The empty vector (EV) control plants are represented by bars positioned to the right of the mutant bars for visual comparison (set as 1). The data are presented as the means ± standard deviations; * indicates p<0.05, ** indicates p<0.01, *** indicates p<0.001, and **** indicates p<0.0001.

## *Supplementary technical methods: Dual-luciferase assay*

The soybean genome database (https://www.soybase.org/) was used to obtain the *GmLAC7, GmLAC12, GmPHT1-4,* and *GmNUD2* gene sequences, primers were designed via the National Center for Biotechnology Information (https://www.ncbi.nlm.nih.gov/) (Table S2), and BamH I and EcoR I restriction sites at both ends of the primers were added. The soybean root cDNA was used as a template, the target fragments were amplified and purified, the pGreenII 0800-miRNA vector was double digested, and the recombinant vector was constructed through homologous recombination. After verification by PCR and sequencing, the recombinant plasmid was transformed into DH5α, which was subsequently extracted and named LUC-GmLAC7, LUC-GmLAC12, LUC-GmPHT1-4, or LUC-GmNUD2.

By mutating the seed region sequence of a miRNA, we prevent its binding to target genes. Single-base or closely spaced multibase mutations are introduced into the plasmid, which are amplified via inverse PCR. The primers used were designed to include a 15–21 bp reverse complementary region and at least a 15 bp noncomplementary region. The target plasmids LUC-GmLAC7, LUC-GmLAC12, LUC-GmPHT1-4, and LUC-GmNUD2 were amplified via the Phanta Max Super-Fidelity DNA Polymerase Kit. After amplification, 5 μL of the product was collected for electrophoresis detection, mixed well and briefly centrifuged. The mixture was incubated at 37 ℃ for 30 minutes and then stored indefinitely at 4 ℃. The recombinant plasmids were transformed into DH5α, the correctness of the results was verified through colony PCR and sequencing, and the resulting plasmids were extracted and named LUC-GmLAC7-MUT, LUC-GmLAC12-MUT, LUC-GmPHT1-4-MUT, and LUC-GmNUD2-MUT.

Five hundred microlitres of successfully transformed Agrobacterium culture was added to 20 mL of LB medium containing 50 μg/mL Kana and 20 μg/mL Rif, and the mixture was cultured at 28 ℃ with shaking at 300 r/min until the OD600 reached approximately 1.0. Then, the mixture was centrifuged at 4500 r/min for 10 min at 4 ℃, the medium was discarded, the bacterial pellet was resuspended in MMA buffer, and the OD600 was adjusted to 0.8 ~ 1.0. The two bacterial cultures were mixed at a 1:1 ratio, and experimental groups were established: miRNA-OE+LUC-Control, miRNA-OE+target gene (wild type), miRNA-OE+target gene (mutant type), EV+LUC-Control, EV+target gene (wild type), and EV+target gene (mutant type). After mixing, the mixture was incubated in the dark for 3 hours, the tobacco leaf was injected with a 10 mL syringe (without a needle), the leaves were marked, the mixture was incubated in the dark for 1 day, and then, the mixture was transferred to normal light conditions for 2--3 days of cultivation.

Three to four tobacco leaves with a diameter of 6 to 8 mm were taken, and holes were punched and placed into a 2 ml EP tube. Then, liquid nitrogen was added for grinding. One hundred microliters of lysis buffer was added to the EP tube, which was incubated on ice for 5 minutes and then centrifuged for 1 minute. Twenty microliters of supernatant was drawn, and this process was repeated three times for each sample. The firefly and Renilla luciferase substrates were diluted to working solutions and incubated at room temperature. The Dual-Glo luciferase assay was performed via a luminometer. 100 microliters of firefly luciferase reaction mixture was added to each well, and the luminescence value was measured. Then, 100 microliters of Renilla luciferase reaction mixture was added to each well, and the luminescence value was measured. The detection should be completed within 30 minutes. Data calculation: Experimental group ratio = (Experimental group F value - Background F value)/(Experimental group R value - Background R value); Control group ratio = (Control group F value - Background F value)/(Control group R value - Background R value); Expression multiple = Experimental group ratio/Control group ratio. Here, F represents the luminescence measurement value of firefly luciferase, R represents the luminescence measurement value of Renilla luciferase, and the blank control group refers to tobacco leaves that have not been injected with bacterial mixture. After injection, the tobacco was treated in the dark for 1 day, cultivated under normal light for 1 day, the leaves were removed, the tobacco was sprayed with D-luciferin potassium salt, the tobacco was left in the dark for 10 minutes, bioluminescence imaging was performed, and images were saved (Toktay et al., 2022).
